# Analysis of Immune Responses in *Acinetobacter baumannii*-Infected Klotho Knockout Mice: A Mouse Model of *Acinetobacter baumannii* Infection in Aged Hosts

**DOI:** 10.3389/fimmu.2020.601614

**Published:** 2020-11-23

**Authors:** Yoshinori Sato, Shigeru Tansho-Nagakawa, Tsuneyuki Ubagai, Yasuo Ono

**Affiliations:** Department of Microbiology and Immunology, Teikyo University School of Medicine, Tokyo, Japan

**Keywords:** *Acinetobacter baumannii*, klotho knockout mice, immune responses, pulmonary inflammation, toll-like receptors

## Abstract

*Acinetobacter baumannii* is an important opportunistic pathogen that primarily afflicts elderly people. To clarify the pathogenicity of *A. baumannii* in the elderly, we investigated immune responses to *A. baumannii* ATCC 19606 infection in klotho knockout (KO) mice, the mouse model of aging. Following intravenous inoculation, the mice seldom displayed severe symptoms. However, the survival rate was 56% at 7 days post-infection. Bacteria were detected in the lungs of klotho KO mice but not klotho wildtype (WT) mice at 7 days post-infection. Neutrophils, eosinophils, interstitial macrophages, and monocyte/dendritic cell subset in the lungs of klotho KO mice were transiently induced after infection with *A. baumannii*. The number of alveolar macrophages in klotho KO mice was lower than that in klotho WT mice, except for 1 day post-infection. CD11b expression on neutrophils and alveolar macrophages in the lungs of klotho KO mice was seldom upregulated by the infection. These results suggested that immune functions eliminating bacteria in the lungs of klotho KO mice were insufficient. CD11b^low^ conventional DC cells hardly increased in klotho KO mice infected with *A. baumannii*. Additionally, the production of interleukin (IL)-10 in the sera of klotho KO mice was significantly higher than that in klotho WT mice, whereas that production of interferon-gamma was not detected in the sera of klotho KO mice. These results suggested that acquired immune responses were hardly induced in klotho KO mice. *IL-1β*, *CXCL1*, *CXCL2*, and *CCL2* expression was significantly higher in the lungs of klotho KO mice infected with *A. baumannii* than in those of klotho WT mice at 1 day post-infection. These results suggested that pulmonary inflammation was elicited in klotho KO mice during early infection. The expression levels of proinflammatory cytokines significantly correlated with *TLR9* expression in the lungs of klotho KO mice. The collective results demonstrate an *A. baumannii* infection state in aged hosts and suggest that pulmonary inflammation and bacterial burden should be noted in aged hosts even in the absence of severe symptoms of *A. baumannii* infection.

## Introduction

*Acinetobacter baumannii* is an important opportunistic pathogen that is associated with nosocomial infections that include bacteremia, pneumonia, meningitis, urinary tract infections, and wound infections (1, 2). The recent increase in multidrug-resistant *A. baumannii* (MDRAB) outbreaks worldwide has become a concern (3–5). Additionally, *A. baumannii* is among the six nosocomial pathogens—*Enterococcus faecium*, *Staphylococcus aureus*, *Klebsiella pneumoniae*, *A. baumannii*, *Pseudomonas aeruginosa*, and *Enterobacter* spp.—that comprise the ESKAPE group that have acquired multidrug resistance and virulence (6, 7). *A. baumannii* is also an emerging pathogen among elderly people in community hospitals and nursing homes (8). Given the accelerating pace of aging of the global population, *A. baumannii* is an important global human pathogen.

Although *A. baumannii* is regarded as a low-virulence pathogen (9), it possesses several mechanisms of pathogenicity, including biofilm formation, adherence, and invasion of lung epithelial cells (10–14), host cell death (15–17), and iron acquisition (18). The pathogenicity of *A. baumannii* depends on various virulence factors, especially the outer membrane proteins (Omps) (19). Additionally, we have reported that MDRAB clinical isolates are resistant to toxicity caused by reactive oxygen species (ROS) in macrophages (20). Taken together, *A. baumannii* is an emerging, highly pathogenic bacterium with characteristics that vary with environmental stress conditions, such as multiple antimicrobial agents and host immune responses. However, only few studies have explored the reasons for the pathogenicity of *A. baumannii* among elderly people.

Some studies on host immune responses to *A. baumannii* infection using mouse models have been performed. Neutrophils and alveolar macrophages (AM) play important roles in host resistance to respiratory infection of *A. baumannii* (21–23). In addition, Toll-like receptor (TLR) 2 and 4 play key roles in eliciting innate immune responses against *A. baumannii* infection (24, 25). TLR9 contributes to the expression of proinﬂammatory cytokines and chemokines (26). These results suggest that innate immune responses play important roles in host resistance to early *A. baumannii* infection. However, these studies have focused on the immune responses of young hosts against *A. baumannii* infection. In contrast, aged mice reportedly showed increased susceptibility to *A. baumannii* infection, increased inflammatory cell infiltration, increased proinflammatory cytokine levels at 24 h post-infection, as well as impaired efficacies of antibiotics and a vaccine (27). These results suggest that immune responses are altered in aged mice compared with the responses in young mice.

Klotho protein is senescence suppressor and its expression naturally reduces in the elderly and in individuals with choric kidney disease (28). Klotho knockout (KO) mice show several aging phenotypes that are similar to human aging (29). These phenotypes include arteriosclerosis, osteoporosis, age-related skin changes, and ectopic calcifications, together with short lifespan and infertility (29). A klotho-septic mouse model that displayed hypercytokinemia with impaired bacterial clearance and increased apoptosis of lymphocytes may be related to poor survival in klotho KO mice treated with cecal ligation and puncture (30). Additionally, a previous study reported that klotho deficiency aggravates sepsis-related multiple organ dysfunction (31). Thus, klotho KO mice are expected to be useful as an animal model for the clarification of pathogenesis of bacterial infections in aged hosts. Generally, aging affects multiple immune systems (32–34). However, although previous studies have reported acute immune responses to *A. baumannii* infection in young mice (35–37), only a few studies have investigated the immune responses of aged hosts to *A. baumannii* infection. Additionally, although immune responses are altered in aged mice compared with those in young mice at 24 h post-infection (27), the immune responses that occur for a few days after *A. baumannii* infection in aged hosts remain unknown.

In this study, we established an *A. baumannii* infection klotho KO mouse model *via* intravenous inoculation and evaluated its immune responses upon infection with *A. baumannii*. Since TLRs play an important role in eliciting immune responses to *A. baumannii* infection (24–26), we also analyzed the correlation between the expression levels of proinflammatory cytokines and those of TLRs in the lungs of the KO mice.

## Materials and Methods

### Bacterial Strains and Growth Condition

ATCC 19606 strains of *A. baumannii* were used in this study. Bacteria were cultured on Luria-Bertani (LB) agar plates (Becton, Dickinson and Company, Sparks, MD, USA) for 16 h at 37°C. The bacteria were washed twice with PBS and resuspended at a concentration of 5 × 10^8^ colony forming units (CFU)/ml, with the concentration adjusted *via* optical density measurements at 595 nm. The bacterial suspensions obtained were used for animal experiments. All procedures were carried out in accordance with the relevant guidelines and regulations.

### Animal Experiments

All animal experiments were performed in accordance with the Institutional Animal Care and Use Committee of Teikyo University Animal Ethics Committee (approval #17–009). Five-week-old male klotho KO mice and littermate klotho wildtype (WT) mice were purchased from CLEA Japan, Inc. (Tokyo, Japan). Bacteria (10^8^ CFU/200 μl) were inoculated intravenously into 6-week-old klotho mice. PBS were inoculated intravenously into 6-week-old klotho mice as uninfected mice. To analyze the number of bacteria in the lungs and spleen of klotho mice, 100 μl of the homogenates in PBS was plated on LB agar plates. The bacterial count (CFU) was confirmed by the growth of serial dilutions of the bacterial suspension on LB agar after 24 h of incubation at 37°C. To analyze the number of bacteria in the blood of klotho mice, 100 μl of blood was plated on LB agar plates. The bacterial count was confirmed using the same method.

### Flow Cytometry Analysis

Cells were harvested from the lungs, spleens, and blood of *A. baumannii*-infected klotho mice and uninfected mice. Briefly, mice were gently killed by cervical dislocation under sedation, and the lungs and spleens were surgically harvested. The lung tissue was cut into small pieces with scissors, and processed in digestion buffer (200 μg/ml of Collagenase Type 1 CLS1 (Worthington Biochemical, Lakewood, NJ) and 2 mM of CaCl_2_ in PBS). The homogenized lungs and spleen were prepared by gently pressing the organs through a 40-μm nylon mesh into a single-cell suspension. The cells were washed and red blood cells were lysed with PharmLyse Lysis Buffer (BD Biosciences, San Jose, CA). The harvested blood was treated with PharmLyse Lysis Buffer (BD) and the cells were washed with PBS. Isolated cells were counted and then stained with monoclonal antibodies (mAbs) for the analysis of immune cells. Briefly, isolated cells from the lungs were stained with various combinations of mAbs. The mAbs included were fluorescein isothiocyanate (FITC)-labeled anti-F4/80, phycoerythrin (PE)-labeled anti-CD11b, PE-Cy-7-labeled anti-CD45, allophycocyanin (APC)-labeled anti-CD11c, and APC-Cy-7-labeled Ly-6G mAbs (Biolegend, San Diego, CA). The white blood cells from the spleen and blood were stained with various combinations of mAbs, including FITC-labeled anti-F4/80, PE-labeled anti-CD11b, PE-Cy-7-labeled anti-Ly-6C, APC-labeled anti-CD11c, and APC-Cy-7-labeled Ly-6G mAbs. To analyze the proportion of immune cells in the lungs, spleen, and blood of klotho mice, immune cells were stained with specific antibodies against surface markers for 30 min at 4°C. The cells were washed twice with stain buffer (BD), and subsequently fixed with BD Cytofix™ Fixation Buffer (BD). The stained cells were analyzed using a FACSCant II flow cytometer equipped with FACS Diva software (BD). All flow cytometry data were analyzed using FlowJo software (BD).

### Cytokine Production

Cytokine levels of tumor necrosis factor-alpha (TNF-α), IL-6, IL-10, IL-12, interferon-gamma (IFN-γ), and chemokine (C-C motif) ligand 2 (CCL2) in the sera of uninfected and *A. baumannii*-infected klotho mice were measured using the Cytometric Bead Array Mouse Inflammation Kit (BD), as per the manufacturer’s protocols. Cytokine levels of mouse chemokine (C-X-C motif) ligand 1 (CXCL1) and CCL7 in the sera of uninfected and *A. baumannii*-infected klotho mice were measured using the Mouse CXCL1 ELISA Kit (Abcam) and the Mouse MCP-3 (CCL7) ELISA Kit (Abcam), as per the manufacturer’s protocols. The lower limits of detection were as follows: TNF-α, 7.3 pg/ml; IL-6, 5 pg/ml; IL-10, 17.5 pg/ml; IL-12p70, 10.7 pg/ml; IFN-γ, 2.5 pg/ml; CXCL1, 5.47 pg/ml; CCL2, 52.7 pg/ml; and CCL7, 6.25 pg/ml.

### RNA Extraction and Quantitative Real-Time Polymerase Chain Reaction (qPCR)

To analyze the expression of proinflammatory cytokines, total RNA was extracted from the lungs of uninfected and *A. baumannii*-infected klotho mice using the RNeasy Plus Mini kit (QIAGEN, Tokyo, Japan). Harvested RNA samples were quantified using the NanoDrop spectrophotometer (Thermo Fisher Scientific, Waltham, MA). Total RNA was reverse-transcribed to cDNA using PrimeScript™ 1^st^ strand cDNA Synthesis Kit (TaKaRa Bio, Shiga, Japan). To analyze the mRNA levels of all genes, cDNA was amplified using the PowerUP SYBR Green Master Mix (Thermo Fisher Scientific) with consensus primers for detecting mouse glyceraldehyde 3-phosphate dehydrogenase (*GAPDH*), mouse *TNF-α*, mouse *IL-1β*, mouse *IL-6*, mouse *IL-10*, mouse *CXCL1*, mouse *CXCL2*, mouse *CCL2*, mouse *CCL7*, mouse *TLR2*, mouse *TLR4*, and mouse *TLR9*. The primer sequences are listed in **Table 1**. Mouse *GAPDH* was used as an internal control to quantify mouse *TNF-α*, mouse *IL-1β*, mouse *IL-6*, mouse *IL-10*, mouse *CXCL1*, mouse *CXCL2*, mouse *CCL2*, mouse *CCL7*, mouse *TLR2*, mouse *TLR4*, and mouse *TLR9*. Real-time PCR was performed using 40 cycles of denaturation at 95°C for 15 s, annealing at 62°C for 15 s, and extension at 72°C for 1 min. The amplified PCR products were quantitatively monitored using a StepOne Real-Time PCR System (Applied Biosystems, Foster City, CA). Fold changes in the expression level of each gene were calculated by the 2^−ΔΔCt^ method using the mouse *GAPDH* gene as an internal control. The relative expression of each gene was evaluated relative to the control sample (uninfected mouse), which was assigned an arbitrary unit value of 1.

**Table 1:** Primers used for real-time PCR.

****Mouse Gene	Sequence****	Reference****
***TNF-α***	F: ATGATCCGCGACGTGGAA R: CTGCCACAAGCAGGAATGAG	(20)
***IL-1β***	F: CGCAGCAGCACATCAACAAGAGC R: TGTCCTCATCCTGGAAGGTCCACG	(20)
***IL-6***	F: CCAGAGATACAAAGAAATGATGG R: ACTCCAGAAGACCAGAGGAAA	(20)
***IL-10***	F: CAGAGCCACATGCTCCTAGA R: GTCCAGCTGGTCCTTTGTTT	(20)
***CXCL1* (*KC*)**	F: CCAGCCACACTCCAACACAGC R: AGGGAGCTTCAGGGTCAAGGC	(38)
***CXCL2* (*MIP-2*)**	F: ACCAACCACCAGGCTACAG R: GCGTCACACTCAAGCTCT	(20)
***CCL2* (*MCP-1*)**	F: TTAAAAACCTGGATCGGAACCAA R: GCATTAGCTTCAGATTTACGGGT	(39)
***CCL7* (*MCP-3*)**	F: TCCACATGCTGCTATGTCAAG R: CATGTCTAAGTATGCTATAGCC	(40)
***TLR2***	F: TGGAATGTCACCAGGCTGC R: GTCCGTGGAAATGGTGGC	(41)
***TLR4***	F: AGGAAGTTTCTCTGGACTAACAAGTTTAGA R: AAATTGTGAGCCACATTGAGTTTC	(41)
***TLR9***	F: TTCTCAAGACGGTGGATCGC R: GCAGAGGGTTGCTTCTCACG	(41)
***GAPDH***	F: CTTCACCACCATGGAGAAGGC R: GGCATGGACTGTGGTCATGAG	(20)

F, forward primer; R, reverse primer.

### Statistical Analyses

Survival curves with log rank test were obtained using EZR (Saitama Medical Center, Jichi Medical University, Saitama, Japan), which is a graphical user interface for R (The R Foundation for Statistical Computing, Vienna, Austria). More precisely, it is a modified version of R commander designed to add statistical functions frequently used in biostatistics (42). Quantitative results were compiled from more than two independent experiments. The results are presented as the mean ± standard error of the mean (SEM). Comparisons of numerical data were performed using the Student’s *t*-test. Pearson correlation analysis was used to compare the mRNA levels of proinflammatory cytokines and *TLR2*, *4*, and *9*. In all analyses, a two-tailed probability of < 5% (i.e., *P < 0.05) was considered statistically significant.

## Results

### Klotho KO Mice Infected With *A. baumannii* Rarely Display Severe Symptoms Prior to Death

**Figure 1:**
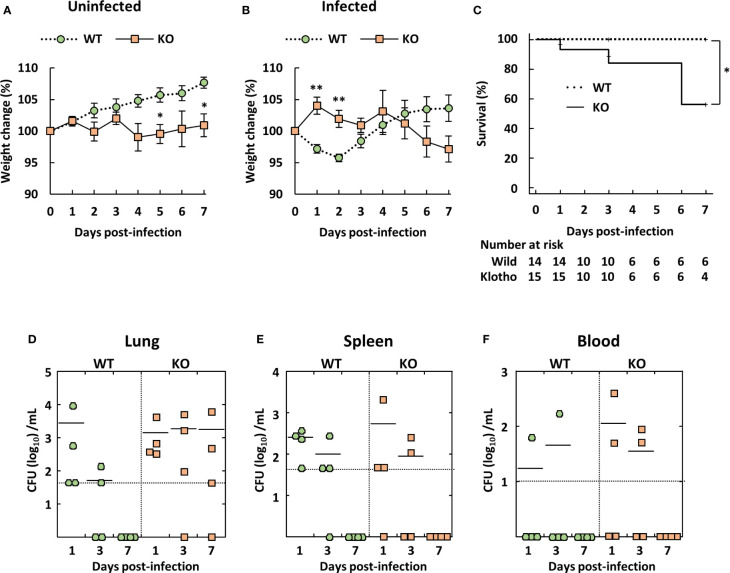
Weight change, survival rate, and bacterial burden of klotho wildtype (WT) and knockout (KO) mice infected with *Acinetobacter baumannii*. *A. baumannii* ATCC 19606 (10^8^ CFU/mouse) was inoculated intravenously into 6-week-old klotho WT and KO mice. **(A)** Summarized results show weight changes of uninfected klotho WT and KO mice, and represent the mean ± SEM (WT, *n* = 5; KO, *n* = 5). **(B)** Summarized results show weight changes of klotho WT and KO mice after infection with *A. baumannii*, and represent the mean ± SEM (WT, *n* = 14; KO, *n* = 15). These data are compiled from three independent experiments. Asterisks indicate statistically significant differences (^**^ *P* < 0.01; ^*^ *P* < 0.05, klotho WT mice *vs.* klotho KO mice; Student’s *t*-test). **(C)** Survival curves of klotho WT and KO mice after infection with *A. baumannii* are shown (WT, *n* = 14; KO, *n* = 15). These data are compiled from three independent experiments. Comparison of survival curves with log rank test yielded statistical significance (^*^ *P* = 0.04). Summarized results show **(D)** bacterial count in the lungs, **(E)** spleen, and **(F)** blood of klotho WT (*n* = 12) and KO (*n* = 12) mice after infection with *A. baumannii*. These data are compiled from two independent experiments. There are no significant differences between klotho WT and KO mice. Each symbol represents one mouse. The mean value is shown as a horizontal solid line.

Young C57BL/6 mice reportedly showed low susceptibility to *A. baumannii* ATCC 19606 infection (35, 36). Therefore, we evaluated body weight, clinical score, and survival rate of klotho WT and KO mice after infection *via* intravenous inoculation of *A. baumannii* ATCC 19606. The body weight of uninfected klotho WT mice gradually increased, whereas that of uninfected klotho KO mice remained stable (**Figure 1A**). Following infection, the body weight of infected klotho WT mice significantly decreased until 2 days post-infection, but had recovered to the pre-infection level at 4 days post-infection. The body weight of klotho KO mice increased at 1 day post-infection and subsequently decreased gradually until 7 days post-infection (**Figure 1B**). A clinical score (ruffled fur) was observed for one of 15 klotho KO mice at 1 day post-infection and one of six klotho KO mice (inactive unless prodded) at 6 days post-infection. The other klotho KO mice infected with *A. baumannii* did not display any clinical symptoms. Additionally, although klotho WT mice did not die of *A. baumannii* infection, klotho KO mice were more susceptible, as evidence by the 56% survival rate at 7 days post-infection (**Figure 1C**). These results suggested that klotho KO mice rarely displayed severe symptoms of *A. baumannii* infection prior to their death. To clarify whether bacteria were eliminated from klotho KO mice, the bacteria in the lungs, spleen, and blood of klotho WT and KO mice were enumerated after infection with *A. baumannii*. Bacteria were not detected in the lungs, spleen, and blood of klotho WT mice at 7 days post-infection (**Figures 1D–F**; WT). In contrast, bacteria were detected in the lungs of three of four klotho KO mice at 7 days post-infection (**Figure 1D**; KO), but not in the spleen and blood (**Figures 1E, F**; KO). These results suggested that *A. baumannii* escaped from host immune responses in the lungs of klotho KO mice and subsequently colonized the lungs of the aged hosts.

### Transient Induction of Neutrophils, Eosinophils, Interstitial Macrophages, and Monocytes/Dendritic Cell (Subset in the Lungs of Klotho KO Mice After Infection With *A. baumannii*

**Figure 2:**
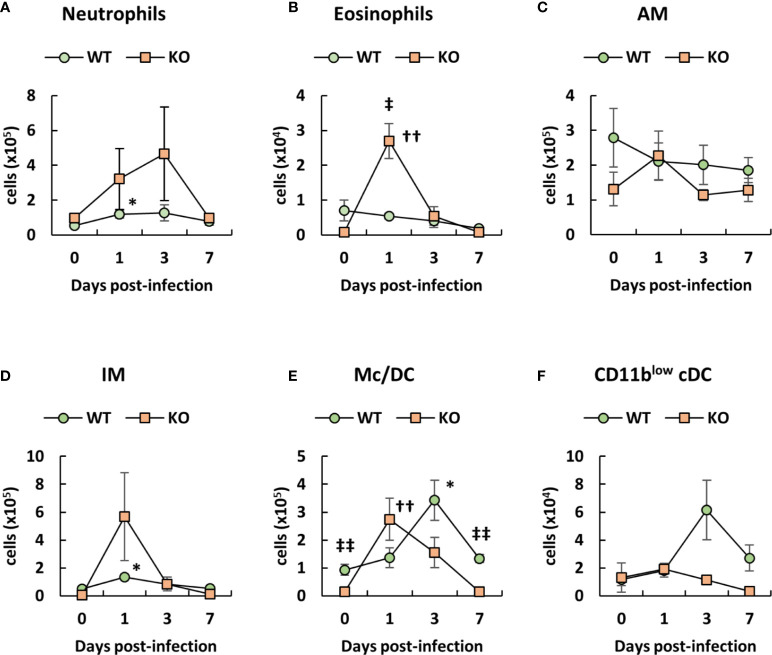
Number of innate immune cells in the lungs of klotho wildtype (WT) and knockout (KO) mice before and after infection with *Acinetobacter baumannii*. Summarized results show the number of **(A)** neutrophils, **(B)** eosinophils, **(C)** alveolar macrophages (AM), **(D)** interstitial macrophages (IM), **(E)** monocytes/dendritic cells (Mc/DC), and **(F)** CD11b^low^ conventional DC (cDC) in the lungs of *A. baumannii*-infected klotho WT and KO mice. Line graphs were compiled from 4 independent experiments (*n* = 16 for WT and *n* = 15 for KO mice), and represent the mean ± SEM. Asterisks indicate statistically significant differences (^*^ *P* < 0.05, klotho WT mice before infection *vs.* klotho WT mice after infection, Student’s *t*-test; ^††^ *P* < 0.01, klotho KO mice before infection *vs.* klotho KO mice after infection, Student’s *t*-test; ^‡‡^ *P* < 0.01, klotho WT mice *vs.* klotho KO mice, Student’s *t*-test).

Since neutrophils and macrophages play an important role in the clearance of *A. baumannii* (21–23), we analyzed the number of immune cells in the lungs of klotho WT and KO mice after infection with *A. baumannii*, based on the classification of the lung innate immune cell population (43–45). Representative results from uninfected and *A. baumannii*-infected klotho mice at 1 day post-infection are shown in **Figures S1** (WT) and **S2** (KO). The results from the two groups are summarized in **Figure 2**. Flow cytometry revealed that the number of neutrophils in the lungs of klotho KO mice gradually increased until 3 days post-infection and decreased at 7 days post-infection, whereas that of klotho WT mice significantly increased at 1 day post-infection (**Figure 2A**). The number of eosinophils in the lungs of klotho KO mice significantly increased at 1 day post-infection and subsequently decreased until 7 days post-infection, whereas klotho WT mice showed no induction of eosinophils after infection (**Figure 2B**). The number of AM in the lungs of klotho KO mice was slightly increased at 1 day post-infection and subsequently decreased to the pre-infection level at 3 days post-infection, whereas the cell number in klotho WT mice slightly decreased after infection (**Figure 2C**). Additionally, the number of AM in klotho KO mice was lower than that in klotho WT mice, except for 1 day post-infection (**Figure 2C**). The number of IMs increased in the lungs of klotho KO mice at 1 day post-infection and subsequently deceased at 3 days post-infection. (**Figure 2D**). The number of CD11b^+/hi^CD11c^−/low^ monocytes plus CD11b^hi^CD11c^low^ Mc/DC subset in the lungs of klotho KO mice increased at 1 day post-infection and subsequently decreased to the pre-infection level by 7 days post-infection, whereas that of klotho WT mice gradually increased until 3 days post-infection and was decreased at 7 days post-infection (**Figure 2E**). The number of CD11b^low^ conventional DC in the lungs of klotho KO mice hardly increased after the infection, whereas that of klotho WT mice increased at 3 days post-infection and subsequently decreased at 7 days post-infection (**Figure 2F**). These results suggested that innate immune cells in the lungs of klotho KO mice were transiently induced after infection of *A. baumannii*. However, this transient change did not eliminate *A. baumannii* in the lungs (**Figure 1D**).

### Negligible Activation of Neutrophils in the Lungs of Klotho KO Mice Infected With *A. baumannii*

**Figure 3:**
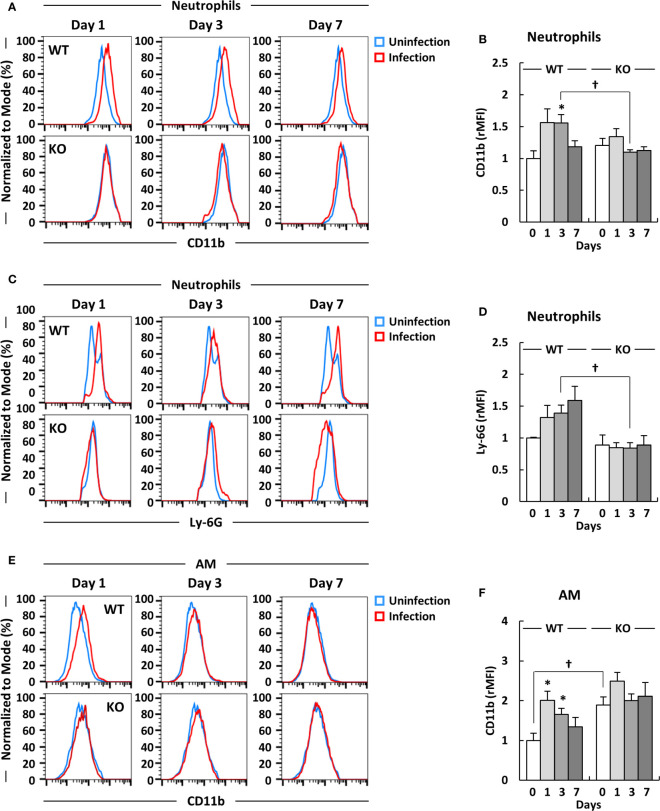
Expression levels of CD11b and Ly-6G on neutrophils, and CD11b on alveolar macrophages (AM) in the lungs of klotho wildtype (WT) and knockout (KO) mice before and after infection with *Acinetobacter baumannii*. Representative results on **(A)** CD11b and **(C)** Ly-6G expression levels on neutrophils and **(E)** CD11b expression level of alveolar macrophages (AM) in the lungs of uninfected and *A. baumannii*-infected klotho WT and KO mice. Mean fluorescence intensity was measured in uninfected (blue line) and infected (red line) klotho mice using flow cytometry. Summarized results on the expression levels of **(B)** CD11b and **(D)** Ly-6G on neutrophils and **(F)** that of CD11b on AM in the lungs of klotho WT and KO mice before and after infection with *A. baumannii*. Bar graph data were compiled from four independent experiments (*n* = 16 for WT and *n* = 15 for KO mice), and represent the mean ± SEM. Asterisks indicate statistically significant differences (^*^ *P* < 0.05, before infection *vs.* after infection, Student’s *t*-test; ^†^ *P* < 0.05, klotho WT mice *vs.* klotho KO mice, Student’s *t*-test).

Previous studies have reported that neutrophils are activated in inflammatory conditions as well as in response to lipopolysaccharide (LPS) stimulation, with the upregulation of markers of the integrin family, such as CD11b, as well as other activation markers (46, 47). Although neutrophils were induced in klotho KO mice after infection with *A. baumannii*, the burden of the lung bacteria was not completely eliminated in the mice at 7 days post-infection. Therefore, we evaluated the expression level of CD11b on neutrophils to determine whether neutrophils were activated in the lungs of klotho KO mice after infection with *A. baumannii*. Representative results from uninfected and *A. baumannii*-infected klotho mice are shown in **Figure 3A** and the complete results are summarized in **Figure 3B**. The expression level of CD11b on neutrophils in the lungs of klotho WT mice was significantly increased after infection with *A. baumannii* (**Figures 3A, B**; WT). In contrast, the expression level of CD11b on neutrophils in the lungs of klotho KO mice was slightly increased at only 1 day post-infection (**Figures 3A, B**; KO). Moreover, there was a significant difference between the expression level of CD11b on neutrophils in the lungs of klotho KO mice and WT mice at 3 days post-infection (**Figure 3B**). In addition, the expression level of Ly-6G as a maturation marker of neutrophils was analyzed in klotho WT and KO mice after infection with *A. baumannii*. The expression level of Ly-6G on neutrophils in the lungs of klotho WT mice gradually increased after the infection, whereas that of klotho KO mice hardly increased after infection with *A. baumannii* (**Figures 3C, D**). There was a significant difference between the expression level of Ly-6G on neutrophils in the lungs of klotho WT mice and KO mice at 3 days post-infection. These results suggest that neutrophils in the lungs of klotho KO mice impair their activation and functional maturation. We further analyzed the expression level of CD11b on AM, because the expression is upregulated by host-derived inflammatory stimuli (48, 49). Representative results from uninfected and *A. baumannii*-infected klotho mice are shown in **Figure 3E** and the collective results are summarized in **Figure 3F**. The expression level of CD11b on AM in the lungs of klotho WT mice was significantly increased at 1 day post-infection and subsequently gradually decreased (**Figures 3E, F**; WT). The expression level of CD11b on AM in the lungs of klotho KO mice before infection was significantly higher than that of klotho WT mice before infection and slightly increased at 1 day post-infection (**Figures 3E, F**; KO). These results suggested that although AM are already activated because of aging, it only slightly responded to *A. baumannii* infection.

### Proinflammatory Cytokines Are Strongly Induced in the Lungs of Klotho KO Mice Infected With *A. baumannii*

**Figure 4:**
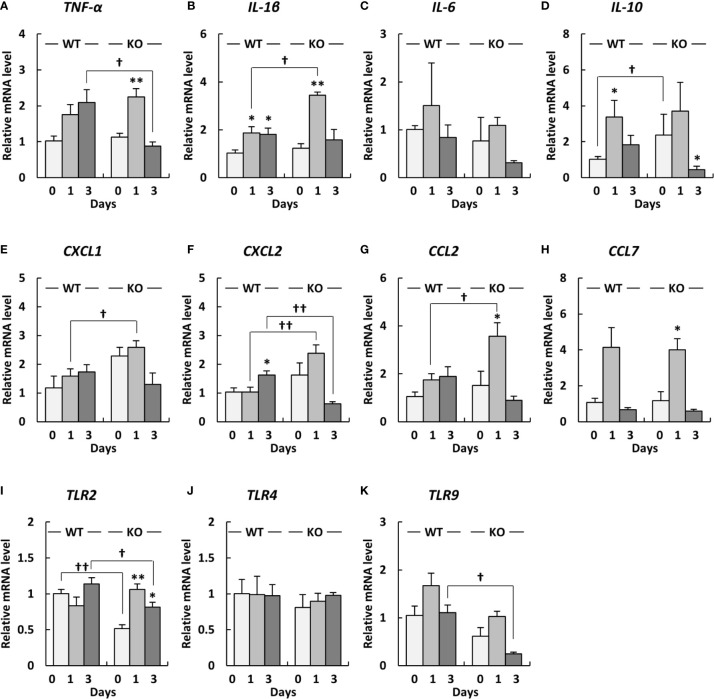
Proinflammatory cytokine expression levels in the lungs and Toll-like receptor (*TLR) 2/4/9* gene expression levels in the lungs of klotho wildtype (WT) and knockout (KO) mice before and after infection with *Acinetobacter baumannii*. The mRNA levels of **(A)** *TNF-α*, **(B)** *IL-1β*, **(C)**, *IL-6* **(D)** *IL-10*, **(E)** *CXCL1*, **(F)** *CXCL2*, **(G)** *CCL2*, **(H)** *CCL7*, **(I)** *TLR2*, **(J)** *TLR4*, and **(K)** *TLR9* in the lungs of klotho WT and KO mice before and after infection with *A. baumannii*. Bar graph data represent the mean ± SEM and were compiled from four independent experiments (*n* = 12 for WT and *n* = 12 for KO mice). Asterisks indicate statistically significant differences (**P < 0.01; *P < 0.05, before infection *vs.* after infection, Student’s *t*-test; ^††^ *P* < 0.01; ^†^ *P* < 0.05, klotho WT mice *vs.* klotho KO mice, Student’s *t*-test).

To investigate inflammatory responses in the lungs of klotho WT and KO mice after infection with *A. baumannii*, we analyzed the mRNA expression of proinflammatory cytokines in the lungs of the mice after infection with *A. baumannii*. The level of *TNF-α* mRNA in the lungs of klotho WT mice gradually increased after infection, whereas those of klotho KO mice significantly increased at 1 day post-infection and subsequently decreased at 3 days post-infection (**Figure 4A**). Additionally, the level of *TNF-α* mRNA in the lungs at 3 days post-infection was significantly lower in klotho KO mice than in klotho WT mice (**Figure 4A**). The level of *IL-1β* mRNA in the lungs of klotho WT mice significantly increased at 1 day post-infection and was maintained at 3 days post-infection, whereas those in the lungs of klotho KO mice strongly and significantly increased at 1 day post-infection and subsequently decreased at 3 days post-infection (**Figure 4B**). The level of *IL-1β* mRNA was significantly higher in the lungs of klotho KO mice than in those of klotho WT mice at 1 day post-infection (**Figure 4B**). The level of *IL-6* mRNA in the lungs of klotho WT mice slightly increased at 1 day post-infection and subsequently deceased at 3 days post-infection (**Figure 4C**). The level of *IL-6* mRNA in klotho KO mice was not increased at 1 day post-infection and was decreased at 3 days post-infection (**Figure 4C**). The level of *IL-10* mRNA before infection was significantly higher in the lungs of klotho KO mice than in those of klotho WT mice (**Figure 4D**). The level of *IL-10* mRNA in the lungs of klotho WT mice significantly increased at 1 day post-infection and decreased at 3 days post-infection (**Figure 4D**). The level of *IL-10* mRNA in the lungs of klotho KO mice increased at 1 day post-infection and subsequently decreased significantly (**Figure 4D**). The level of *CXCL1* mRNA in the lungs of klotho WT mice gradually increased after infection (**Figure 4E**). The level of *CXCL1* mRNA in the lungs before infection was higher in klotho KO mice than in klotho WT mice (**Figure 4E**). Additionally, the level of *CXCL1* mRNA in the lungs at 1 day post-infection was significantly higher in klotho KO mice than in klotho WT mice (**Figure 4E**). The level of *CXCL2* mRNA in the lungs of klotho KO mice increased at 1 day post-infection and subsequently decreased at 3 days post-infection (**Figure 4F**). The level of *CXCL2* mRNA was significantly higher in the lungs of klotho KO mice than in those of klotho WT mice at 1 day post-infection (**Figure 4F**). The level of *CCL2* mRNA in the lungs of klotho WT mice gradually increased after infection, whereas those of klotho KO mice significantly increased at 1 day post-infection and subsequently decreased at 3 days post-infection (**Figure 4G**). The level of *CCL2* mRNA in the lungs at 1 day post-infection was significantly higher in klotho KO mice than in klotho WT mice (**Figure 4G**). The level of *CCL7* mRNA increased in the lungs of klotho WT and KO mice at 1 day post-infection and subsequently decreased at 3 days post-infection (**Figure 4H**). The expression levels of *CCL7* mRNA were comparable between klotho WT and KO mice after infection (**Figure 4H**). The collective results suggested that proinflammatory responses were strongly and transiently induced in the lungs of klotho KO mice after infection with *A. baumannii*.

### Expression Levels of Proinflammatory Cytokines Correlate With Those of *TLR9* in the Lungs of Klotho KO Mice

**Figure 5:**
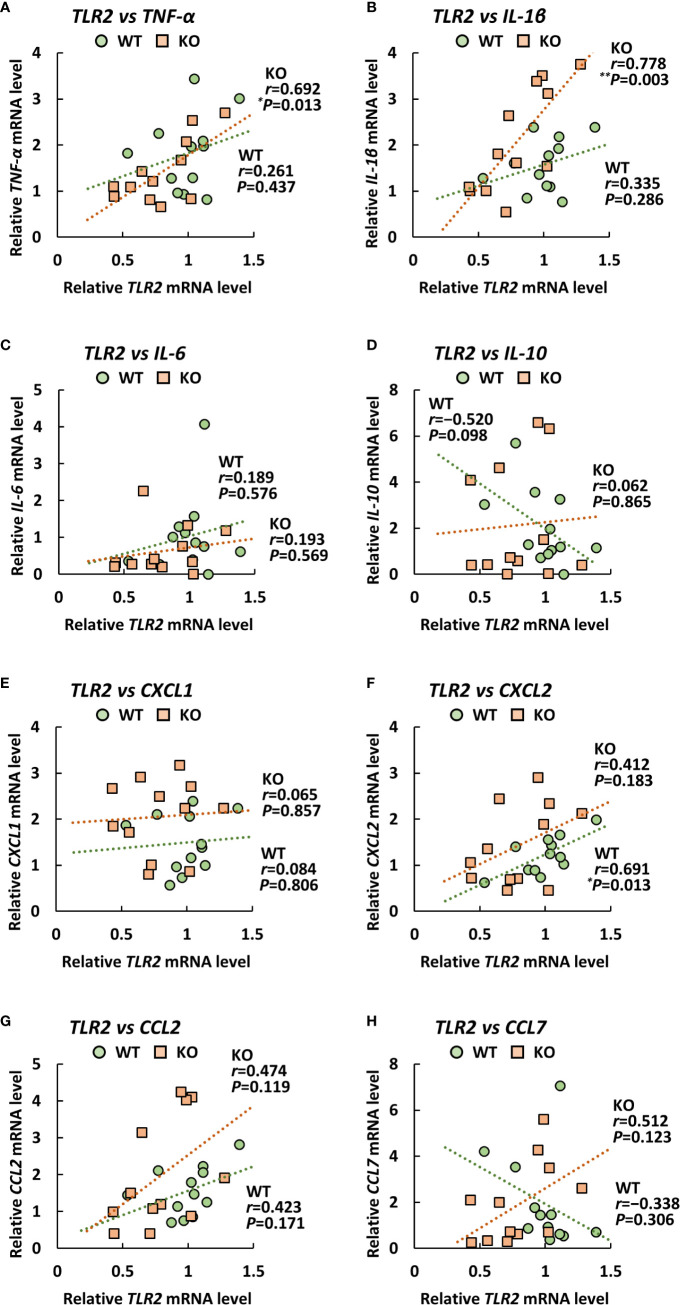
Relationship between the expression of proinflammatory cytokines and Toll-like receptor 2 (*TLR2*) in klotho wildtype (WT) and knockout (KO) mice. Pearson correlation coefficient was calculated between the mRNA level of *TLR2* and that of **(A)** *TNF-α*, **(B)** *IL-1β*, **(C)**, *IL-6* **(D)** *IL-10*, **(E)** *CXCL1*, **(F)** *CXCL2*, **(G)** *CCL2*, **(H)** *CCL7* in klotho WT and KO mice. Each symbol represents one mouse. Asterisks indicate statistically significant correlations (**P < 0.01; *P < 0.05).

**Figure 6:**
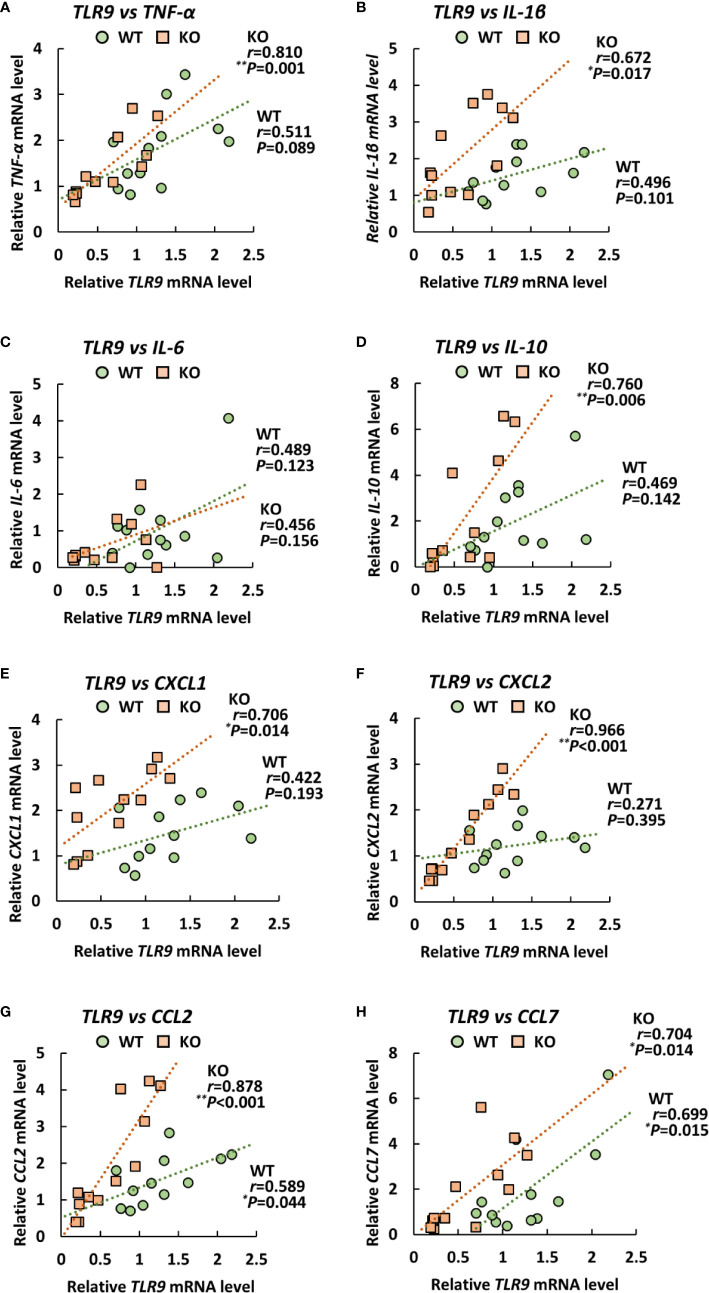
Relationship between the expression of proinflammatory cytokines and Toll-like receptor 9 (*TLR9*) in klotho wildtype (WT) and knockout (KO) mice. Pearson correlation coefficient was calculated between the mRNA level of *TLR9* and that of **(A)** *TNF-α*, **(B)** *IL-1β*, **(C)**, *IL-6* **(D)** *IL-10*, **(E)** *CXCL1*, **(F)** *CXCL2*, **(G)** *CCL2*, **(H)** *CCL7* in klotho WT and KO mice. Each symbol represents one mouse. Asterisks indicate statistically significant correlations (**P < 0.01; *P < 0.05).

Since TLRs play an important role in eliciting proinflammatory cytokines during infection with *A. baumannii* (24–26), we analyzed the expression levels of *TLR2*, *4*, and *9* in the lungs of klotho WT and KO mice after infection with *A. baumannii*. The level of *TLR2* mRNA was significantly lower in the lungs of uninfected KO mice than in those of uninfected klotho WT mice (**Figure 4I**). Following infection, the level of *TLR2* mRNA in the lungs of klotho WT mice was stable but was significantly increased in KO mice at 1 and 3 days post-infection. The level of *TLR2* mRNA was significantly lower in the lungs of klotho KO mice than in those of klotho WT mice at 3 days post-infection. In addition, the level of *TLR9* mRNA was lower in the lungs of uninfected KO mice than in those of klotho WT mice (**Figure 4K**). Following infection, the levels of *TLR9* mRNA in the lungs of klotho WT and KO mice increased at 1 day post-infection and subsequently decreased at 3 days post-infection. However, the level of *TLR9* mRNA in the lungs of klotho KO mice was significantly decreased compared with that of klotho WT mice at 3 days post-infection. In contrast, the levels of *TLR4* mRNA were comparable between klotho WT and KO mice, and were not altered in their lungs after the infection (**Figure 4J**). We further analyzed the correlation between the expression levels of proinflammatory cytokines and those of TLRs in the lungs of klotho WT and KO mice after infection with *A. baumannii*. Pearson correlation analysis revealed that the mRNA level of *CXCL2* was significantly correlated with that of *TLR2* in the lungs of klotho WT mice (**Figure 5F**). In contrast, mRNA levels of *TNF-α* and *IL-1β* were significantly correlated with those of *TLR2* in the lungs of klotho KO mice (**Figures 5A, B**). Additionally, mRNA levels of *IL-6, IL-10, CXCL1, CCL2*, and *CCL7* were not correlated with those of *TLR2* in the lungs of klotho WT and KO mice (**Figures 5C–E, G, H**). Moreover, mRNA levels of *CCL2* and *CCL7* were significantly correlated with those of *TLR9* in the lungs of klotho WT mice (**Figures 6G, H**). In contrast, the mRNA levels of *TNF-α*, *IL-1β*, *IL-10* *CXCL1, CXCL2, CCL2, and CCL7* were strongly and significantly correlated with those of *TLR9* in the lungs of klotho KO mice (**Figures 6A, B, D–H**). Additionally, mRNA level of *IL-6* was not correlated with that of *TLR9* in the lungs of klotho WT and KO mice (**Figure 6C**). These results indicated the important roles of TLR9 in eliciting proinflammatory responses in the lungs of aged hosts against *A. baumannii* infection.

### Different Immune Cell Induction Pattern in the Blood of Klotho KO Mice After Infection With *A. baumannii*

**Figure 7:**
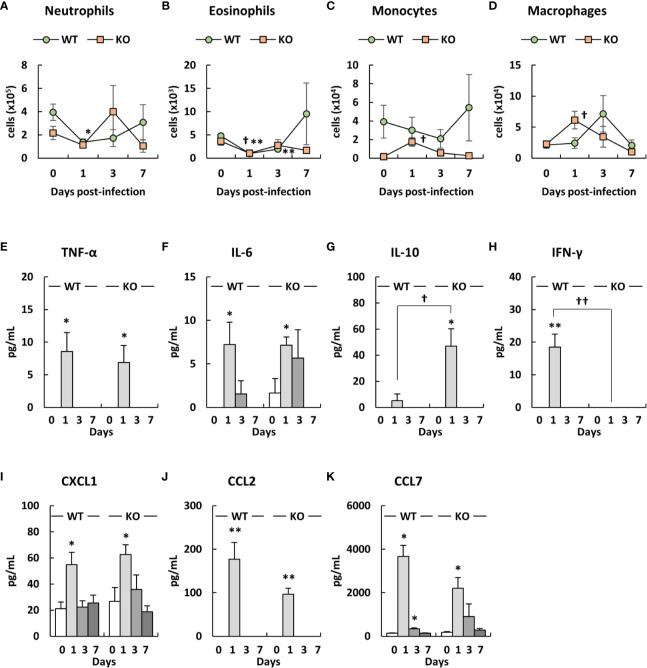
Number of innate immune cells in the blood and proinflammatory cytokine expression levels in the sera of klotho wildtype (WT) and knockout (KO) mice before and after infection with *Acinetobacter baumannii*. Summarized results show the number of **(A)** neutrophils, **(B)** eosinophils, **(C)** monocytes, and **(D)** macrophages in the blood of *A. baumannii*-infected klotho WT and KO mice. Line graphs were compiled from four independent experiments (*n* = 16 for WT and *n* = 16 for KO mice), and represent the mean ± SEM. Asterisks indicate statistically significant differences (^*^ *P* < 0.05, klotho WT mice before infection *vs.* klotho WT mice after infection, Student’s *t*-test; ^††^ *P* < 0.01, klotho KO mice before infection *vs.* klotho KO mice after infection, Student’s *t*-test; ^‡‡^ *P* < 0.01, klotho WT mice *vs.* klotho KO mice, Student’s *t*-test). The production of **(E)** TNF-α, **(F)** IL-6, **(G)** IL-10, **(H)** IFN-γ, **(I)** CXCL1, **(J)** CCL2, and **(K)** CCL7 in the sera of klotho WT and KO mice before and after infection with *A. baumannii*. Bar graph data represent the mean ± SEM and were compiled from four independent experiments (*n* = 16 for WT and *n* = 16 for KO mice). Asterisks indicate statistically significant differences (**P < 0.01; *P < 0.05, before infection *vs.* after infection, Student’s *t*-test; ^††^ *P* < 0.01; ^†^ *P* < 0.05, klotho WT mice *vs.* klotho KO mice, Student’s *t*-test).

We analyzed the number of immune cells in the blood of klotho WT and KO mice after infection with *A. baumannii*. Representative results from uninfected and *A. baumannii*-infected klotho mice at 1 day post-infection are shown in **Figures S3** (WT) and **S4** (KO). The results from the two groups are summarized in **Figures 7A–D**. Flow cytometry revealed that the number of neutrophils in the blood before infection was lower in klotho KO mice than in klotho WT mice (**Figure 7A**). The number of neutrophils in the blood of klotho WT mice significantly decreased at 1 day-post infection and subsequently increased gradually (**Figure 7A**). In contrast, the number of neutrophils in the blood of klotho KO mice increased at 3 days post-infection and subsequently decreased at 7 days post-infection (**Figure 7A**). The number of eosinophils significantly decreased in the blood of klotho WT and KO mice at 1 day-post infection and subsequently increased (**Figure 7B**). The number of monocytes in the blood of klotho KO mice significantly increased at 1 day post-infection and subsequently decreased at 3 days post-infection (**Figure 7C**). However, the number of monocytes in the blood before and after infection was lower in klotho KO mice than in klotho WT mice (**Figure 7C**). The number of macrophages in the blood of klotho KO mice significantly increased at 1 day post-infection and subsequently decreased gradually, whereas those of klotho WT mice increased at 3 day post-infection and subsequently decreased at 7 days post-infection (**Figure 7D**).

### Klotho KO Mice Infected With *A. baumannii* Display Different Cytokine Production Pattern in Their Sera

We analyzed the levels of proinflammatory cytokines in the sera of klotho WT and KO mice before and after infection with *A. baumannii*. TNF-α, IL-6, CXCL1, CCL2, and CCL7 levels significantly increased in the sera of klotho WT and KO mice at 1 day post-infection, while the level of IL-12 was not (**Figures 7E, F, I–K**). Of note, although the serum level of IL-6, CXCL1, and CCL7 in klotho WT mice decreased at 3 days post-infection, those in klotho KO mice was maintained until 3 days post-infection (**Figures 7F, I, K**). The serum level of IL-10 increased in klotho WT and KO mice after infection and was significantly higher in klotho KO mice than in klotho WT mice at 1 day post-infection (**Figure 7G**). The serum level of IFN-γ significantly increased in klotho WT mice at 1 day post-infection, whereas that did not increase in infected klotho KO mice (**Figure 7H**). These results suggested that proinflammatory responses were prolonged in the periphery of klotho KO mice infected with *A. baumannii*.

## Discussion

*A. baumannii* has recently emerged as a major nosocomial pathogen (1, 2). Although we have previously reported the virulence characteristics of *A. baumannii* clinical isolates in human epithelial cells and macrophages *in vitro* (13, 14, 20), the pathogenicity of *A. baumannii* infection in elderly people has remained elusive. In the current study, we established an aged mouse model of *A. baumannii* infection using klotho KO mice and evaluated the immune responses in the mice before and after infection.

Previous study reported an aged pneumonia mouse model by non-invasive intratracheal inoculation with *A. baumannii* LAC-4 strain (27). Higher mortality was observed in aged mice along with increased bacterial burdens and more severe lung injury. In addition, increased inflammatory cell infiltration and enhanced pro-inflammatory cytokines at 24 h post-infection were detected in aged mice. *A. baumannii* LAC-4 strain is much more virulent than *A. baumannii* ATCC 19606 strain (36). As *A. baumannii* ATCC 19606 strain was used in this study, klotho KO mice seldom displayed severe symptoms of *A. baumannii* infection but 44% died. Moreover, bacteria were not completely eliminated in the lungs of these mice. These results suggest that *A. baumannii* increases its bacterial burdens and inflammatory cell infiltration in the lungs of aged hosts and can eventually lead to death. Neutrophils and AM play important roles in the clearance of *A. baumannii* (21–23). Although the number of neutrophils increased in the lungs of klotho KO mice after infection with *A. baumannii*, the expression level of CD11b on neutrophils was hardly upregulated. Previous studies have reported that the expression level of CD11b on neutrophils is upregulated by LPS stimulation *via* TLR4 (46, 47). However, signal transduction and functional changes were observed in neutrophils with aging (50). Considering the above findings, our results suggest that TLR4-mediated signaling was weak in the neutrophils of klotho KO mice. The age-related disruption in the function and/or differentiation of these cells in klotho KO mice resulted in a bacterial burden in the lungs of the mice. Additionally, there were fewer AM in klotho KO mice than in klotho WT mice, except for 1 day post-infection. The level of expression of CD11b on AM in the lungs of uninfected klotho KO mice was significantly higher than that of uninfected klotho WT mice. Previous studies demonstrated the upregulated expression of CD11b on AM because of initial inflammation or LPS stimulation (48, 49). However, another study reported that the expression of CD11b is upregulated in AM in old mice and the cells are significantly better in phagocytosing *Mycobacterium tuberculosis* than the CD11b^−^ AM population, which enhanced bacterial growth (51). These results suggest *M. tuberculosis* phagocytosis and survival are enhanced in aged hosts. Likewise, *A. baumannii* resist toxicity caused by ROS and survive in macrophages (20). Considering the above findings, the phagocytosis of *A. baumannii* by AM protects the bacteria from the lethal action of neutrophils and allows the bacteria to establish an intractable infection in aged hosts. Conversely, there were more IMs in the lungs of klotho KO mice infected with *A. baumannii*. A previous study reported that IMs are induced by acute lung injury caused by, for instance, high doses of LPS, and perform important immune functions, including the maintenance of lung homeostasis (52). These results suggest that acute inflammation is elicited in the lungs of aged hosts early during *A. baumannii* infection.

Inflammatory monocytes are recruited into tissues and kill pathogens *via* phagocytosis, ROS activity, production of nitric oxide (NO), and the action of inflammatory cytokines (53, 54). Although we analyzed ROS production in monocytes as well as neutrophils in klotho KO mice infected with *A. baumannii*, it was not clear whether those cells induced ROS in response to the infection. Further studies are required to clarify the production of ROS in those cells. On the other hands, inflammatory monocytes upregulate levels of CD11c and MHC class II, which can promote T cell proliferation (53). In the present study, inflammatory monocytes in the lungs, spleen (**Figures S5**, **S6**, and **S7**), and blood (**Figures 7**, **S3**, and **S4**) of klotho KO mice infected with *A. baumannii* were transiently induced. However, the number of inflammatory monocytes in those tissues were lower in klotho KO mice than in klotho WT mice. These results suggest that *A. baumannii* infection is complicated by the low induction of inflammatory monocytes in aged hosts. Additionally, klotho KO mice infected with *A. baumannii* showed lower induction of DC subsets than infected klotho WT mice (**Figures 2** and **S7**). These results suggest that age-associated changes in the proportion of monocyte and DC subsets affect adaptive immune function, thereby reducing the efficacies of antibiotics and vaccines in aged hosts (27).

Although the present study suggests the disruption in the function and/or differentiation of immune cells in klotho KO mice, it remains unclear how klotho KO mice died from infection with *A. baumannii*. A previous study reported that klotho deficiency aggravates sepsis-related multiple organ dysfunction (31). Thus, one possible reason for eventually death might be incomplete bacteria clearance and worsen multiple organ dysfunction. Hearps *et al*. reported that monocytes from older individuals exhibit impaired phagocytosis but contain shortened telomeres, suggesting a dysregulation of monocytes function in the aged hosts (55). Moreover, it is suggested that telomere shortening in peripheral monocytes reflects telomere shortening in haematopoietic bone marrow precursor cells (55). Considering the above findings, impaired activation of immune cells in klotho KO mice may be ascribed to telomere shortening in haematopoietic bone marrow precursor cells and causes incomplete bacteria clearance in the lungs, and then led to death with sepsis-related multiple organ dysfunction.

IL-1β is a potent proinflammatory cytokine. Its overexpression can lead to tissue damage (56). For instance, decreased IL-1β in inflammatory responses leads to a better outcome in acute lung infections with *Pseudomonas aeruginosa* (57). Pulmonary inflammation in mice after infection with *A. baumannii* has been associated with IL-1β production and the severity of lung pathology (58). In addition, CC and CXC chemokines attract neutrophils and mononuclear cells to sites of inflammation (59–62). Mediators of inflammation, including IL-1 and TNF-α, or bacterial products such as LPS elicit the production of those chemokines (59). Presently, the induction level of *IL-1β*, *CXCL1* (*KC*), *CXCL2* (*MIP-2*), and *CCL2* (*MCP-1*) mRNA in the lungs after infection was significantly higher in klotho KO mice than in klotho WT mice. This result suggests that klotho KO mice infected with *A. baumannii* showed severe lung inflammation compared with klotho WT mice, which sometimes led to death. A previous study reported that TLR2 KO mice displayed increased lung concentrations of MCP-1 and MIP-2 after infection with *A. baumannii* (24). The authors discussed the possibility that TLR2 mediates anti-inflammatory pathways that downregulate MCP-1 production. We also demonstrated that the expression level of *TLR2* mRNA was lower in the lungs of uninfected klotho KO mice than in those of uninfected klotho WT mice. However, the expression level in the lungs of klotho KO mice was upregulated at 1 day post infection of *A. baumannii*. These results suggest the possibility that initial infection of *A. baumannii* in aged hosts causes severe inflammation. Moreover, the expression level of *TLR9* mRNA in the lungs of klotho KO mice was lower than that of klotho WT mice. TLR9 KO mice challenged intranasally with *A. baumannii* reportedly displayed a significantly increased bacterial burden and neutrophil recruitment in the lungs, with reduced levels of proinflammatory cytokines, such as TNF-α, IFN-γ, and MCP-1, in their sera during systemic *A. baumannii* infection (26). These phenotypes are similar to those of klotho KO mice infected with *A. baumannii* in the present study. Additionally, we demonstrated that the expression of proinflammatory cytokines was strongly and significantly correlated with that of *TLR9* mRNA in the lungs of klotho KO mice. The above findings suggest that TLR9-mediated immune responses may play an important role in the lung inflammation of elderly people with *A. baumannii* infection.

In this study, lung bacterial burden remained in klotho KO mice at 7 days post-infection in spite of the infection established by the intravenous inoculation of *A. baumannii*. Previous studies have demonstrated that the virulence factors of *A. baumannii*, such as OmpA and Bap, mediate bacterial adherence to bronchial and lung epithelial cells (11, 12). Additionally, we have reported that the levels of *ompA* expression in MDRAB clinical isolates correlate with the adherence of the bacteria to lung epithelial cells (13). We also reported a novel bacterial transport mechanism, where *A. baumannii* exploits human neutrophils by adhering to the cells (63). These results support the novel idea concerning *A. baumannii* infection in elderly people, in which the bacteria infect aged hosts *via* the peripheral blood, respiratory system, and other routes, followed by the movement with neutrophils to the lung tissue and subsequent colonization of the lungs. Additionally, the production of proinflammatory cytokines in the sera after infection with *A. baumannii* were prolonged in klotho KO mice compared with klotho WT mice. However, although klotho WT mice showed decreased weight after infection with *A. baumannii*, klotho KO mice showed only a slight weight change after the infection. Additionally, most of klotho KO mice infected with *A. baumannii* did not display any clinical symptoms. These results suggest that weak inflammation lasting for prolonged periods are observed in aged hosts.

A previous study reported that *A. baumannii* infection inhibits airway eosinophilia and lung pathology in a mouse model of allergic asthma (64). Our results indicate that eosinophils are decreased in the lungs of klotho WT mice after infection with *A. baumannii*, implying the inhibition of airway eosinophilia in young hosts with allergic asthma. However, the significant increase in eosinophils in the lungs of klotho KO mice after infection with *A. baumannii* indicates further induction of airway eosinophilia in elderly people with allergic asthma. Thus, the pulmonary inflammation in *A. baumannii* infection in elderly people may be a different clinical condition compared with that in young people.

In summary, our results suggest that *A. baumannii* infection in aged hosts involves accumulation of the bacteria in the lungs, which elicits pulmonary inflammation during early infection and can eventually lead to death. The results also suggest that pulmonary inflammation correlates with altered TLR9 expression in klotho KO mice. This study provides insights into the pathogenicity of *A. baumannii* infection, particularly in elderly people. Further studies are required to understand the mechanism of *A. baumannii* infection in elderly people.

## Data Availability Statement

The original contributions presented in the study are included in the article/supplementary material. Further inquiries can be directed to the corresponding author.

## Ethics Statement

The animal study was reviewed and approved by Institutional Animal Care and Use Committee of Teikyo University Animal Ethics Committee (no. 1705236A1b).

## Author Contributions

Conceptualization, data curation, formal analysis, investigation, visualization, writing—original draft preparation: YS. Funding acquisition, project administration, supervision: YS, YO. Methodology: YS, ST-N, TU. Resources, validation, writing—review and editing: YS, ST-N, TU, YO. All authors contributed to the article and approved the submitted version.

## Funding

This research was supported by JSPS KAKENHI Grant Number 17K15692, 17K10032, and 20K08827.

## Conflict of Interest

The authors declare that the research was conducted in the absence of any commercial or financial relationships that could be construed as a potential conflict of interest.

## Supplementary Material

Supplementary Figure 1 Phenotypic analysis of innate immune cells in the lungs of *Acinetobacter baumannii*-infected and uninfected klotho wildtype (WT) mice. Innate immune cells in the lungs of *A. baumannii*-infected and uninfected klotho WT mice were analyzed for their expression of the following cell-surface markers: CD45, CD11b, CD11c, Ly-6G, and F4/80. The results represent 4-color flow cytometric analysis of the innate immune cell population in lung homogenates from **(A)** an uninfected klotho WT mouse and **(B)** an *A. baumannii*-infected mouse at 1 day post-infection. Np, Neutrophils (CD45^+^CD11b^+^Ly-6G^+^); AM, Alveolar macrophages (CD45^+^CD11b^low^CD11c^high^Ly-6G^−^F4/80^+^); IM, Interstitial macrophages (CD45^+^CD11b^high^CD11c^low/−^Ly-6G^−^F4/80^+^FSC^mid/high^SSC^mid^); Mc, Monocytes (CD45^+^CD11b^+^CD11c^−^Ly-6G^−^F4/80^low/−^); Mc/DC, Monocytes/dendritic cells (CD45^+^CD11b^high^CD11c^+^Ly-6G^−^F4/80^low/−^); Ep, Eosinophils (CD45^+^CD11b^high^CD11c^low/−^Ly-6G^−^F4/80^+^FSC^mid^SSC^high^); CD11b^low^ cDC, CD11b^low^ conventional dendritic cells (CD45^+^CD11b^low^CD11c^+^Ly-6G^−^F4/80^low/−^).

Supplementary Figure 2 Phenotypic analysis of innate immune cells in the lungs of *Acinetobacter baumannii*-infected and uninfected klotho knockout (KO) mice. Innate immune cells in the lungs of *A. baumannii*-infected and uninfected klotho KO mice were analyzed for their expression of the following cell-surface markers: CD45, CD11b, CD11c, Ly-6G, and F4/80. A representative result of 4-color flow cytometric analysis of the innate immune cell population in lung homogenates from **(A)** an uninfected klotho KO mouse and **(B)** an *A. baumannii*-infected mouse at 1 day post-infection. Np, Neutrophils (CD45^+^CD11b^+^Ly-6G^+^); AM, Alveolar macrophages (CD45^+^CD11b^low^CD11c^high^Ly-6G^−^F4/80^+^); IM, Interstitial macrophages (CD45^+^CD11b^high^CD11c^low/−^Ly-6G^−^F4/80^+^FSC^high/mid^SSC^mid^); Mc, Monocytes (CD45^+^CD11b^+^CD11c^−^Ly-6G^−^F4/80^low/−^); Mc/DC, Monocytes/dendritic cells (CD45^+^CD11b^high^CD11c^+^Ly-6G^−^F4/80^low/−^); Ep, Eosinophils (CD45^+^CD11b^high^CD11c^low/−^Ly-6G^−^F4/80^+^FSC^mid^SSC^high^); CD11b^lo^ cDC, CD11b^lo^ conventional dendritic cells (CD45^+^CD11b^low^CD11c^+^Ly-6G^−^F4/80^low/−^).

Supplementary Figure 3 Phenotypic analysis of innate immune cells in the blood of *Acinetobacter baumannii*-infected and uninfected klotho wildtype (WT) mice. Innate immune cells in the blood of *A. baumannii*-infected and uninfected klotho WT mice were analyzed for their expression of the following cell-surface markers: CD11b, CD11c, Ly-6C, Ly-6G, and F4/80. A representative result of 4-color flow cytometric analysis of the innate immune cell population in blood cells from **(A)** an uninfected klotho WT mouse and **(B)** an *A. baumannii*-infected mouse at 1 day post-infection. Np, Neutrophils (CD11b^+^Ly-6G^+^); MΦ, Macrophages (CD11b^high/+^CD11c^−^Ly-6C^+/−^Ly-6G^−^F4/80^+^ FSC^high/mid^SSC^mid/lo^); Mc, Monocytes (CD11b^high/+^CD11c^lo/−^Ly-6C^high^Ly-6G^−^F4/80^+^FSC^high/mid^SSC^mid/lo^); Ep, Eosinophils (CD11b^high/+^CD11c^−^Ly-6G^−^FSC^mid^SSC^high^); DC, Dendritic cells (CD11b^low/−^CD11c^high^Ly-6G^−^).

Supplementary Figure 4 Phenotypic analysis of innate immune cells in the blood of *Acinetobacter baumannii*-infected and uninfected klotho knockout (KO) mice. Innate immune cells in the blood of *A. baumannii*-infected and uninfected klotho KO mice were analyzed for their expression of the following cell-surface markers: CD11b, CD11c, Ly-6C, Ly-6G, and F4/80. A representative result of 4-color flow cytometric analysis of the innate immune cell population in blood cells from **(A)** an uninfected klotho WT mouse and **(B)** an *A. baumannii*-infected mouse at 1 day post-infection. Np, Neutrophils (CD11b^+^Ly-6G^+^); MΦ, Macrophages (CD11b^high/+^CD11c^−^Ly-6C^+/−^Ly-6G^−^F4/80^+^ FSC^high/mid^SSC^mid/lo^); Mc, Monocytes (CD11b^high/+^CD11c^lo/−^Ly-6C^high^Ly-6G^−^F4/80^+^FSC^high/mid^SSC^mid/lo^); Ep, Eosinophils (CD11b^high/+^CD11c^−^Ly-6G^−^FSC^mid^SSC^high^); DC, Dendritic cells (CD11b^low/−^CD11c^high^Ly-6G^−^).

Supplementary Figure 5 Phenotypic analysis of innate immune cells in the spleen of *Acinetobacter baumannii*-infected and uninfected klotho wildtype (WT) mice. Innate immune cells in the spleen of *A. baumannii*-infected and uninfected klotho WT mice were analyzed for their expression of the following cell-surface markers: CD11b, CD11c, Ly-6C, Ly-6G, and F4/80. A representative result of 4-color flow cytometric analysis of the innate immune cell population in splenocytes from **(A)** an uninfected klotho WT mouse and **(B)** an *A. baumannii*-infected mouse at 1 day post-infection. Np, Neutrophils (CD11b^+^Ly-6G^+^); MΦ, Macrophages (CD11b^high/+^CD11c^−^Ly-6C^+/−^Ly-6G^−^F4/80^+^ FSC^high/mid^SSC^mid/lo^); Mc, Monocytes (CD11b^high/+^CD11c^lo/−^Ly-6C^high^Ly-6G^−^F4/80^+^FSC^high/mid^SSC^mid/lo^); Ep, Eosinophils (CD11b^high/+^CD11c^−^Ly-6G^−^FSC^mid^SSC^high^); DC, Dendritic cells (CD11b^low/−^CD11c^high^Ly-6G^−^).

Supplementary Figure 6 Phenotypic analysis of innate immune cells in the spleen of *Acinetobacter baumannii*-infected and uninfected klotho knockout (KO) mice. Innate immune cells in the spleen of *A. baumannii*-infected and uninfected klotho KO mice were analyzed for their expression of the following cell-surface markers: CD11b, CD11c, Ly-6C, Ly-6G, and F4/80. A representative result of 4-color flow cytometric analysis of the innate immune cell population in splenocytes from **(A)** an uninfected klotho WT mouse and **(B)** an *A. baumannii*-infected mouse at 1 day post-infection. Np, Neutrophils (CD11b^+^Ly-6G^+^); MΦ, Macrophages (CD11b^high/+^CD11c^−^Ly-6C^+/−^Ly-6G^−^F4/80^+^ FSC^high/mid^SSC^mid/lo^); Mc, Monocytes (CD11b^high/+^CD11c^lo/−^Ly-6C^high^Ly-6G^−^F4/80^+^FSC^high/mid^SSC^mid/lo^); Ep, Eosinophils (CD11b^high/+^CD11c^−^Ly-6G^−^FSC^mid^SSC^high^); DC, Dendritic cells (CD11b^low/−^CD11c^high^Ly-6G^−^).

Supplementary Figure 7 Number of innate immune cells in the spleen of klotho wildtype (WT) and knockout (KO) mice before and after infection with *Acinetobacter baumannii*. Summarized results show the number of **(A)** neutrophils, **(B)** eosinophils, **(C)** monocytes, **(D)** macrophages, and **(E)** dendritic cells (DC) in the spleen of *A. baumannii*-infected klotho WT and KO mice. Line graphs were compiled from 4 independent experiments (*n* = 16 for WT and *n* = 16 for KO mice), and represent the mean ± SEM. Asterisks indicate statistically significant differences as follows: ^**^ *P* < 0.01, ^*^ *P* < 0.05, klotho WT mice before infection *vs.* klotho WT mice after infection, Student’s *t*-test; ^††^ *P* < 0.01, ^†^ *P* < 0.05, klotho KO mice before infection *vs.* klotho KO mice after infection, Student’s *t*-test; and ^‡‡^ *P* < 0.01, ^‡^ *P* < 0.05, klotho WT mice *vs.* klotho KO mice, Student’s *t*-test).

## References

[1] Fournier PE Richet H The Epidemiology and Control of Acinetobacter baumannii in Health Care Facilities. Clin Infect Dis (2006) 42:692–9. 10.1086/500202 16447117

[2] Munoz-Price LS Weinstein RA *Acinetobacter* infection. N Engl J Med (2008) 358:1271–81. 10.1056/NEJMra070741 18354105

[3] Kempf M Rolain JM Emergence of resistance to carbapenems in *Acinetobacter baumannii* in Europe: clinical impact and therapeutic options. Int J Antimicrob Agents (2012) 39:105–14. 10.1016/j.ijantimicag.2011.10.004 22113193

[4] Antunes LC Visca P Towner KJ *Acinetobacter baumannii*: evolution of a global pathogen. Pathog Dis (2014) 71:292–301. 10.1111/2049-632X.12125 24376225

[5] Ushizawa H Yahata Y Endo T Iwashima T Misawa M Sonobe M A Epidemiological Investigation of a Nosocomial Outbreak of Multidrug-Resistant *Acinetobacter baumannii* in a Critical Care Center in Japan, 2011-2012. Jpn J Infect Dis (2016) 69:143–8. 10.7883/yoken.JJID.2015.049 26073736

[6] Rice LB Federal funding for the study of antimicrobial resistance in nosocomial pathogens: no ESKAPE. J Infect Dis (2008) 197:1079–81. 10.1086/533452 18419525

[7] Mulani MS Kamble EE Kumkar SN Tawre MS Pardesi KR Emerging Strategies to Combat ESKAPE Pathogens in the Era of Antimicrobial Resistance: A Review. Front Microbiol (2019) 10:539. 10.3389/fmicb.2019.00539 30988669 PMC6452778

[8] Sengstock DM Thyagarajan R Apalara J Mira A Chopra T Kaye KS Multidrug-resistant *Acinetobacter baumannii*: an emerging pathogen among older adults in community hospitals and nursing homes. Clin Infect Dis (2010) 50:1611–16. 10.1086/652759 20462357

[9] Peleg AY Seifert H Paterson DL *Acinetobacter baumannii*: emergence of a successful pathogen. Clin Microbiol Rev (2008) 21:538–82. 10.1128/CMR.00058-07 PMC2493088 18625687

[10] Gaddy JA Tomaras AP Actis LA The *Acinetobacter baumannii* 19606 OmpA protein plays a role in biofilm formation on abiotic surfaces and in the interaction of this pathogen with eukaryotic cells. Infect Immun (2009) 77:3150–60. 10.1128/IAI.00096-09 PMC2715673 19470746

[11] Brossard KA Campagnari AA The *Acinetobacter baumannii* biofilm-associated protein plays a role in adherence to human epithelial cells. Infect Immun (2012) 80:228–33. 10.1128/IAI.05913-11 PMC3255684 22083703

[12] Smani Y McConnell MJ Pachón J Role of fibronectin in the adhesion of *Acinetobacter baumannii* to host cells. PloS One (2012) 7:e33073. 10.1371/journal.pone.0033073 22514602 PMC3326023

[13] Sato Y Unno Y Kawakami S Ubagai T Ono Y Virulence characteristics of *Acinetobacter baumannii* clinical isolates vary with the expression levels of *omps* . J Med Microbiol (2017) 66:203–12. 10.1099/jmm.0.000394 27902395

[14] Sato Y Unno Y Ubagai T Ono Y Sub-minimum inhibitory concentrations of colistin and polymyxin B promote *Acinetobacter baumannii* biofilm formation. PloS One (2018) 13:e0194556. 10.1371/journal.pone.0194556 29554105 PMC5858813

[15] Choi CH Lee EY Lee YC Park TI Kim HJ Hyun SH Outer membrane protein 38 of *Acinetobacter baumannii* localizes to the mitochondria and induces apoptosis of epithelial cells. Cell Microbiol (2005) 7:1127–38. 10.1111/j.1462-5822.2005.00538.x 16008580

[16] Choi CH Hyun SH Lee JY Lee JS Lee YS Kim SA *Acinetobacter baumannii* outer membrane protein A targets the nucleus and induces cytotoxicity. Cell Microbiol (2008) 10:309–19. 10.1111/j.1462-5822.2007.01041.x 17760880

[17] Jin JS Kwon SO Moon DC Gurung M Lee JH Kim SI *Acinetobacter baumannii* secretes cytotoxic outer membrane protein A *via* outer membrane vesicles. PloS One (2011) 6:e17027. 10.1371/journal.pone.0017027 21386968 PMC3046175

[18] Zimbler DL Penwell WF Gaddy JA Menke SM Tomaras AP Connerly PL Iron acquisition functions expressed by the human pathogen *Acinetobacter baumannii* . Biometals (2009) 22:23–32. 10.1007/s10534-008-9202-3 19130255

[19] Li FJ Starrs L Burgio G Tug of war between *Acinetobacter baumannii* and host immune responses. Pathog Dis (2018) 76:ftz004. 10.1093/femspd/ftz004 30657912

[20] Sato Y Unno Y Miyazaki C Ubagai T Ono Y Multidrug-resistant *Acinetobacter baumannii* resists reactive oxygen species and survives in macrophages. Sci Rep (2019) 9:17462. 10.1038/s41598-019-53846-3 31767923 PMC6877552

[21] van Faassen H KuoLee R Harris G Zhao X Conlan JW Chen W Neutrophils play an important role in host resistance to respiratory infection with *Acinetobacter baumannii* in mice. Infect Immun (2007) 75:5597–608. 10.1128/IAI.00762-07 PMC2168347 17908807

[22] Qiu H KuoLee R Harris G Van Rooijen N Patel GB Chen W Role of macrophages in early host resistance to respiratory *Acinetobacter baumannii* infection. PloS One (2012) 7:e40019. 10.1371/journal.pone.0040019 22768201 PMC3386929

[23] Lee HH Aslanyan L Vidyasagar A Brennan MB Tauber MS Carrillo-Sepulveda MA Depletion of alveolar macrophages increases pulmonary neutrophil infiltration, tissue damage, and sepsis in a murine model of *Acinetobacter baumannii* pneumonia. Infect Immun (2020) 88:e00128–20. 10.1128/IAI.00128-20 PMC7309625 32366576

[24] Knapp S Wieland CW Florquin S Pantophlet R Dijkshoorn L Tshimbalanga N Differential roles of CD14 and toll-like receptors 4 and 2 in murine *Acinetobacter* pneumonia. Am J Respir Crit Care Med (2006) 173:122–9. 10.1164/rccm.200505-730OC 16210672

[25] Kim CH Kim DJ Lee SJ Jeong YJ Kang MJ Lee JY Toll-like receptor 2 promotes bacterial clearance during the initial stage of pulmonary infection with *Acinetobacter baumannii* . Mol Med Rep (2014) 9:1410–4. 10.3892/mmr.2014.1966 24567035

[26] Noto MJ Boyd KL Burns WJ Varga MG Peek RM Jr Skaar EP Toll-Like Receptor 9 Contributes to Defense against *Acinetobacter baumannii* Infection. Infect Immun (2015) 83:4134–41. 10.1128/IAI.00410-15 PMC4567622 26238713

[27] Gu H Liu D Zeng X Peng LS Yuan Y Chen ZF Aging exacerbates mortality of *Acinetobacter baumannii* pneumonia and reduces the efficacies of antibiotics and vaccine. Aging (Albany NY) (2018) 10:1597–608. 10.18632/aging.101495 PMC6075437 30018181

[28] Kurosu H Yamamoto M Clark JD Pastor JV Nandi A Gurnani P Suppression of aging in mice by the hormone Klotho. Science (2005) 309:1829–33. 10.1126/science.1112766 PMC2536606 16123266

[29] Kuro-o M Matsumura Y Aizawa H Kawaguchi H Suga T Utsugi T Mutation of the mouse klotho gene leads to a syndrome resembling ageing. Nature (1997) 390:45–51. 10.1038/36285 9363890

[30] Inoue S Sato T Suzuki-Utsunomiya K Komori Y Hozumi K Chiba T Sepsis-induced hypercytokinemia and lymphocyte apoptosis in aging-accelerated *Klotho* knockout mice. Shock (2013) 39:311–6. 10.1097/SHK.0b013e3182845445 23364432

[31] Jorge LB Coelho FO Sanches TR Malheiros DMAC Ezaquiel de Souza L Dos Santos F Klotho deficiency aggravates sepsis-related multiple organ dysfunction. Am J Physiol Renal Physiol (2019) 316:F438–48. 10.1152/ajprenal.00625.2017 30516423

[32] Weng NP Aging of the immune system: how much can the adaptive immune system adapt? Immunity (2006) 24:495–9. 10.1016/j.immuni.2006.05.001 PMC2266981 16713964

[33] Kovacs EJ Palmer JL Fortin CF Fülöp T Jr Goldstein DR Linton PJ Aging and innate immunity in the mouse: impact of intrinsic and extrinsic factors. Trends Immunol (2009) 30:319–24. 10.1016/j.it.2009.03.012 PMC2898122 19541536

[34] Montgomery RR Shaw AC Paradoxical changes in innate immunity in aging: recent progress and new directions. J Leukoc Biol (2015) 98:937–43. 10.1189/jlb.5MR0315-104R PMC4661037 26188078

[35] López-Rojas R Domínguez-Herrera J McConnell MJ Docobo-Peréz F Smani Y Fernández-Reyes M Impaired virulence and in vivo fitness of colistin-resistant *Acinetobacter baumannii* . J Infect Dis (2011) 203:545–8. 10.1093/infdis/jiq086 PMC3071218 21216865

[36] Harris G Kuo Lee R Lam CK Kanzaki G Patel GB Xu HH A mouse model of *Acinetobacter baumannii*-associated pneumonia using a clinically isolated hypervirulent strain. Antimicrob Agents Chemother (2013) 57:3601–13. 10.1128/AAC.00944-13 PMC3719758 23689726

[37] Bruhn KW Pantapalangkoor P Nielsen T Tan B Junus J Hujer KM Host fate is rapidly determined by innate effector-microbial interactions during *Acinetobacter baumannii* bacteremia. J Infect Dis (2015) 211:1296–305. 10.1093/infdis/jiu593 PMC4447835 25378635

[38] Song AQ Gao B Fan JJ Zhu YJ Zhou J Wang YL NLRP1 inflammasome contributes to chronic stress-induced depressive-like behaviors in mice. J Neuroinflamm (2020) 17:178. 10.1186/s12974-020-01848-8 PMC7281929 32513185

[39] Liu H Liu Z Chen J Chen L He X Zheng R Induction of CCL8/MCP-2 by mycobacteria through the activation of TLR2/PI3K/Akt signaling pathway. PloS One (2013) 8:e56815. 10.1371/journal.pone.0056815 23418602 PMC3572057

[40] Auray G Lacroix-Lamandé S Mancassola R Dimier-Poisson I Laurent F Involvement of intestinal epithelial cells in dendritic cell recruitment during *C. parvum* infection. Microbes Infect (2007) 9:574–82. 10.1016/j.micinf.2007.01.026 17395519

[41] Johnson AC Heinzel FP Diaconu E Sun Y Hise AG Golenbock D Activation of toll-like receptor (TLR)2, TLR4, and TLR9 in the mammalian cornea induces MyD88-dependent corneal inflammation. Invest Ophthalmol Vis Sci (2005) 46:589–95. 10.1167/iovs.04-1077 15671286

[42] Kanda Y Investigation of the freely available easy-to-use software ‘EZR’ for medical statistics. Bone Marrow Transplant (2013) 48:452–8. 10.1038/bmt.2012.244 PMC3590441 23208313

[43] Misharin AV Morales-Nebreda L Mutlu GM Budinger GR Perlman H Flow cytometric analysis of macrophages and dendritic cell subsets in the mouse lung. Am J Respir Cell Mol Biol (2013) 49:503–10. 10.1165/rcmb.2013-0086MA PMC3824047 23672262

[44] Yu YR O’Koren EG Hotten DF Kan MJ Kopin D Nelson ER A Protocol for the Comprehensive Flow Cytometric Analysis of Immune Cells in Normal and Inflamed Murine Non-Lymphoid Tissues. PloS One (2016) 11:e0150606. 10.1371/journal.pone.0150606 26938654 PMC4777539

[45] Hoffmann F Ender F Schmudde I Lewkowich IP Köhl J König P Origin, Localization, and Immunoregulatory Properties of Pulmonary Phagocytes in Allergic Asthma. Front Immunol (2016) 7:107. 10.3389/fimmu.2016.00107 27047494 PMC4803735

[46] Andonegui G Bonder CS Green F Mullaly SC Zbytnuik L Raharjo E Endothelium-derived Toll-like receptor-4 is the key molecule in LPS-induced neutrophil sequestration into lungs. J Clin Invest (2003) 111:1011–20. 10.1172/JCI16510 PMC152584 12671050

[47] Lakschevitz FS Hassanpour S Rubin A Fine N Sun C Glogauer M Identification of neutrophil surface marker changes in health and inflammation using high-throughput screening flow cytometry. Exp Cell Res (2016) 342:200–9. 10.1016/j.yexcr.2016.03.007 26970376

[48] Kirby AC Raynes JG Kaye PM CD11b regulates recruitment of alveolar macrophages but not pulmonary dendritic cells after pneumococcal challenge. J Infect Dis (2006) 193:205–13. 10.1086/498874 16362884

[49] Duan M Steinfort DP Smallwood D Hew M Chen W Ernst M CD11b immunophenotyping identifies inflammatory profiles in the mouse and human lungs. Mucosal Immunol (2016) 9:550–63. 10.1038/mi.2015.84 PMC7101582 26422753

[50] Fulop T Larbi A Douziech N Fortin C Guérard KP Lesur O Signal transduction and functional changes in neutrophils with aging. Aging Cell (2004) 3:217–26. 10.1111/j.1474-9728.2004.00110.x 15268755

[51] Lafuse WP Rajaram MVS Wu Q Moliva JI Torrelles JB Turner J Identification of an Increased Alveolar Macrophage Subpopulation in Old Mice That Displays Unique Inflammatory Characteristics and Is Permissive to Mycobacterium tuberculosis Infection. J Immunol (2019) 203:2252–64. 10.4049/jimmunol.1900495 PMC6783358 31511357

[52] Schyns J Bureau F Marichal T Lung Interstitial Macrophages: Past, Present, and Future. J Immunol Res (2018) 2018:5160794. 10.1155/2018/5160794 29854841 PMC5952507

[53] Serbina NV Jia T Hohl TM Pamer EG Monocyte-mediated defense against microbial pathogens. Annu Rev Immunol (2008) 26:421–52. 10.1146/annurev.immunol.26.021607.090326 PMC2921669 18303997

[54] Yang J Zhang L Yu C Yang XF Wang H Monocyte and macrophage differentiation: circulation inflammatory monocyte as biomarker for inflammatory diseases. Biomark Res (2014) 2:1. 10.1186/2050-7771-2-1 24398220 PMC3892095

[55] Hearps AC Martin GE Angelovich TA Cheng WJ Maisa A Landay AL Aging is associated with chronic innate immune activation and dysregulation of monocyte phenotype and function. Aging Cell (2012) 11:867–75. 10.1111/j.1474-9726.2012.00851.x 22708967

[56] Mariathasan S Monack DM Inflammasome adaptors and sensors: intracellular regulators of infection and inflammation. Nat Rev Immunol (2007) 7:31–40. 10.1038/nri1997 17186029

[57] Wonnenberg B Bischoff M Beisswenger C Dinh T Bals R Singh B The role of IL-1β in Pseudomonas aeruginosa in lung infection. Cell Tissue Res (2016) 364:225–9. 10.1007/s00441-016-2387-9 26984603

[58] Kang MJ Jo SG Kim DJ Park JH NLRP_3_ inflammasome mediates interleukin-1β production in immune cells in response to *Acinetobacter baumannii* and contributes to pulmonary inflammation in mice. Immunology (2017) 150:495–505. 10.1111/imm.12704 28032341 PMC5343352

[59] Charo IF Ransohoff RM The many roles of chemokines and chemokine receptors in inflammation. N Engl J Med (2006) 354:610–21. 10.1056/NEJMra052723 16467548

[60] Serbina NV Pamer EG Monocyte emigration from bone marrow during bacterial infection requires signals mediated by chemokine receptor CCR2. Nat Immunol (2006) 7:311–7. 10.1038/ni1309 16462739

[61] Sadik CD Kim ND Luster AD Neutrophils cascading their way to inflammation. Trends Immunol (2011) 32:452–60. 10.1016/j.it.2011.06.008 PMC3470857 21839682

[62] Sanz MJ Kubes P Neutrophil-active chemokines in *in vivo* imaging of neutrophil trafficking. Eur J Immunol (2012) 42:278–83. 10.1002/eji.201142231 22359100

[63] Kamoshida G Tansho-Nagakawa S Kikuchi-Ueda T Nakano R Hikosaka K Nishida S A novel bacterial transport mechanism of *Acinetobacter baumannii via* activated human neutrophils through interleukin-8. J Leukoc Biol (2016) 100:1405–12. 10.1189/jlb.4AB0116-023RR 27365529

[64] Qiu H Kuolee R Harris G Zhou H Miller H Patel GB *Acinetobacter baumannii* infection inhibits airway eosinophilia and lung pathology in a mouse model of allergic asthma. PloS One (2011) 6:e22004. 10.1371/journal.pone.0022004 21789200 PMC3138758

