# Supplementary figures and images for: Analysis of Immune Responses in Acinetobacter baumannii-Infected Klotho Knockout Mice: A Mouse Model of Acinetobacter baumannii Infection in Aged Hosts
Source: Front Immunol. 2020 Nov 23;11:601614. doi: 10.3389/fimmu.2020.601614 (PMC7719750; doi:10.3389/fimmu.2020.601614)

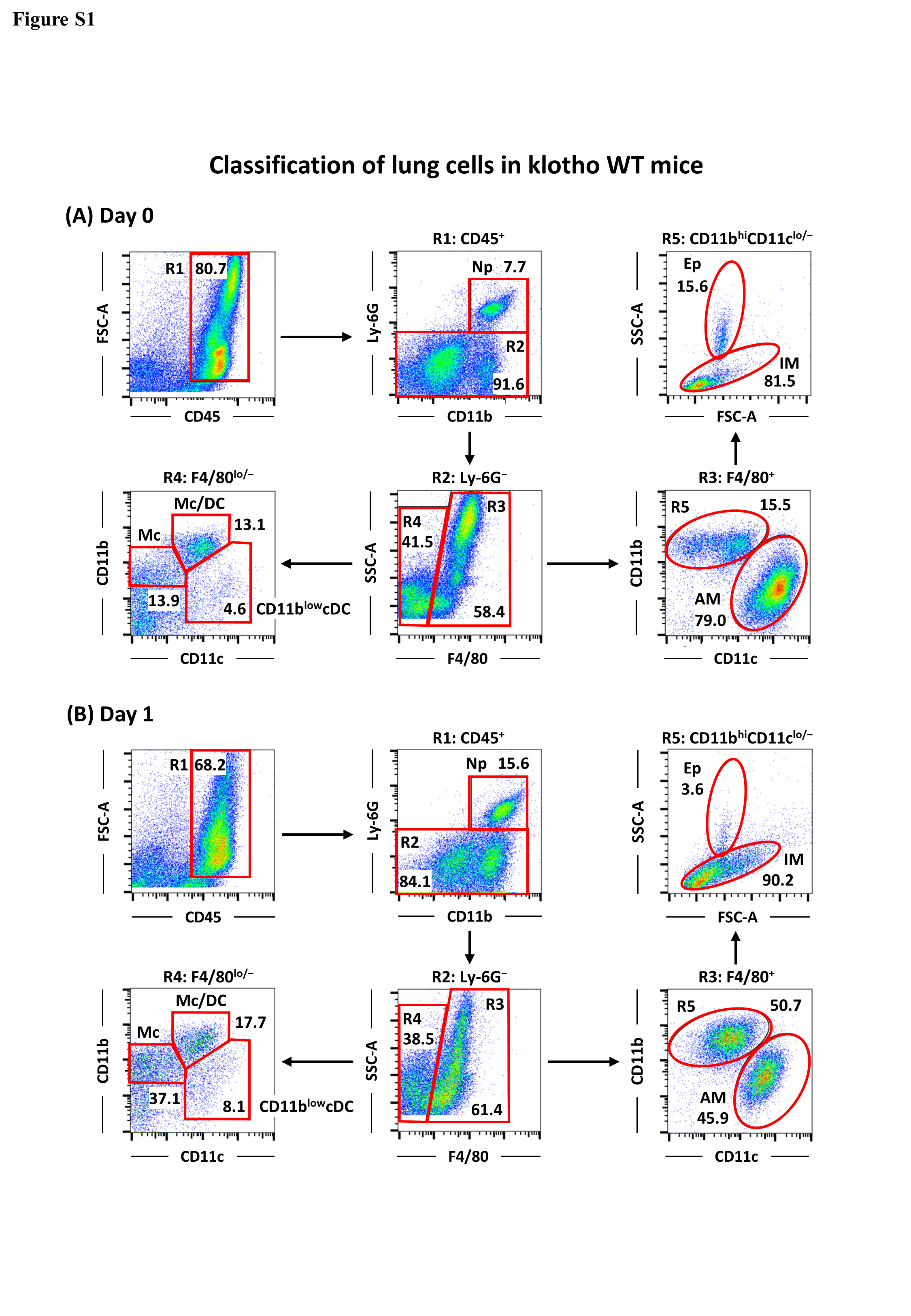

Supplement: Supplementary Figure 1 — Phenotypic analysis of innate immune cells in the lungs of Acinetobacter baumannii-infected and uninfected klotho wildtype (WT) mice. Innate immune cells in the lungs of A. baumannii-infected and uninfected klotho WT mice were analyzed for their expression of the following cell-surface markers: CD45, CD11b, CD11c, Ly-6G, and F4/80. The results represent 4-color flow cytometric analysis of the innate immune cell population in lung homogenates from (A) an uninfected klotho WT mouse and (B) an A. baumannii-infected mouse at 1 day post-infection. Np, Neutrophils (CD45+CD11b+Ly-6G+); AM, Alveolar macrophages (CD45+CD11blowCD11chighLy-6G−F4/80+); IM, Interstitial macrophages (CD45+CD11bhighCD11clow/−Ly-6G−F4/80+FSCmid/highSSCmid); Mc, Monocytes (CD45+CD11b+CD11c−Ly-6G−F4/80low/−); Mc/DC, Monocytes/dendritic cells (CD45+CD11bhighCD11c+Ly-6G−F4/80low/−); Ep, Eosinophils (CD45+CD11bhighCD11clow/−Ly-6G−F4/80+FSCmidSSChigh); CD11blow cDC, CD11blow conventional dendritic cells (CD45+CD11blowCD11c+Ly-6G−F4/80low/−). [file Image_1.tif]

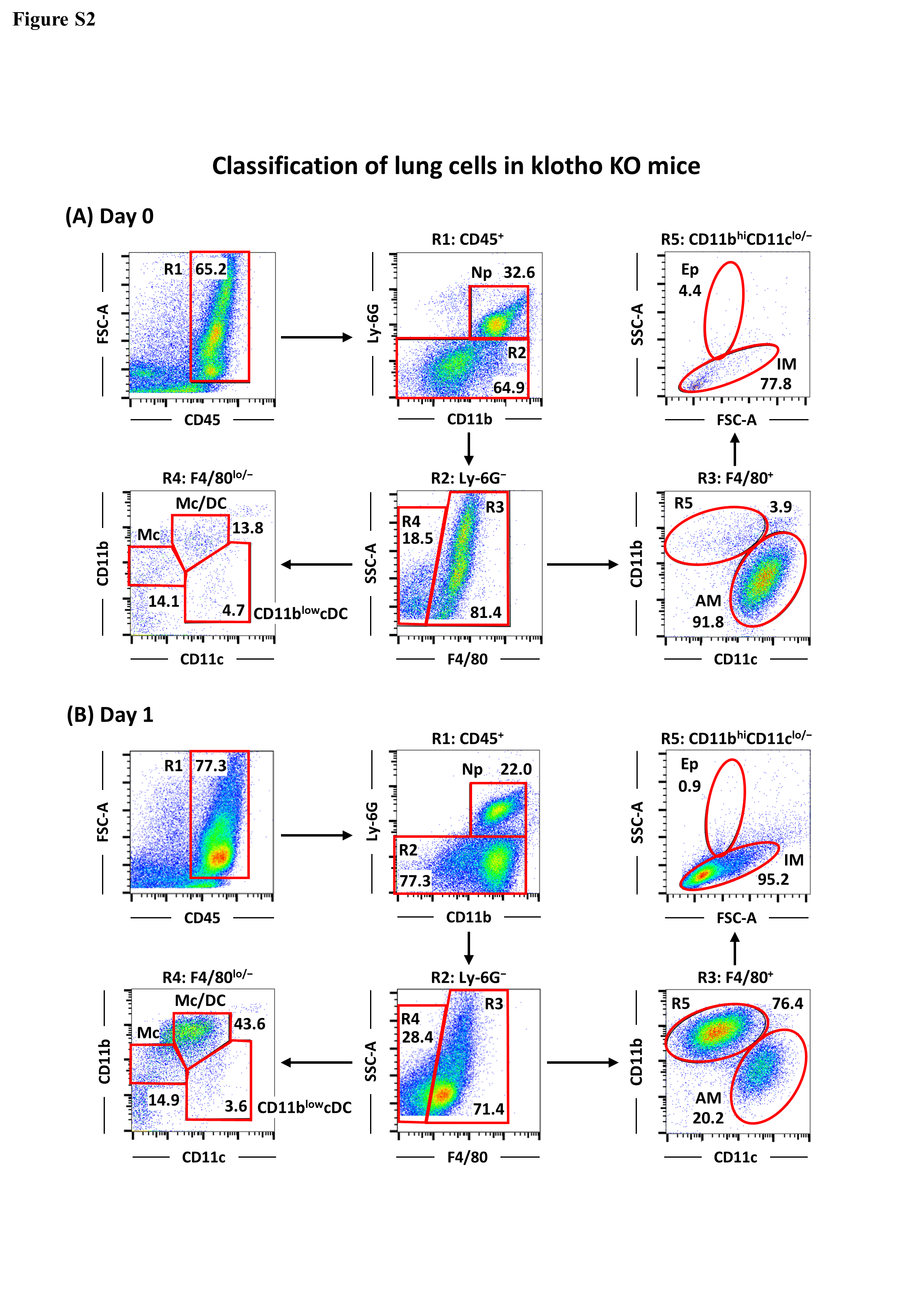

Supplement: Supplementary Figure 2 — Phenotypic analysis of innate immune cells in the lungs of Acinetobacter baumannii-infected and uninfected klotho knockout (KO) mice. Innate immune cells in the lungs of A. baumannii-infected and uninfected klotho KO mice were analyzed for their expression of the following cell-surface markers: CD45, CD11b, CD11c, Ly-6G, and F4/80. A representative result of 4-color flow cytometric analysis of the innate immune cell population in lung homogenates from (A) an uninfected klotho KO mouse and (B) an A. baumannii-infected mouse at 1 day post-infection. Np, Neutrophils (CD45+CD11b+Ly-6G+); AM, Alveolar macrophages (CD45+CD11blowCD11chighLy-6G−F4/80+); IM, Interstitial macrophages (CD45+CD11bhighCD11clow/−Ly-6G−F4/80+FSChigh/midSSCmid); Mc, Monocytes (CD45+CD11b+CD11c−Ly-6G−F4/80low/−); Mc/DC, Monocytes/dendritic cells (CD45+CD11bhighCD11c+Ly-6G−F4/80low/−); Ep, Eosinophils (CD45+CD11bhighCD11clow/−Ly-6G−F4/80+FSCmidSSChigh); CD11blo cDC, CD11blo conventional dendritic cells (CD45+CD11blowCD11c+Ly-6G−F4/80low/−). [file Image_2.tif]

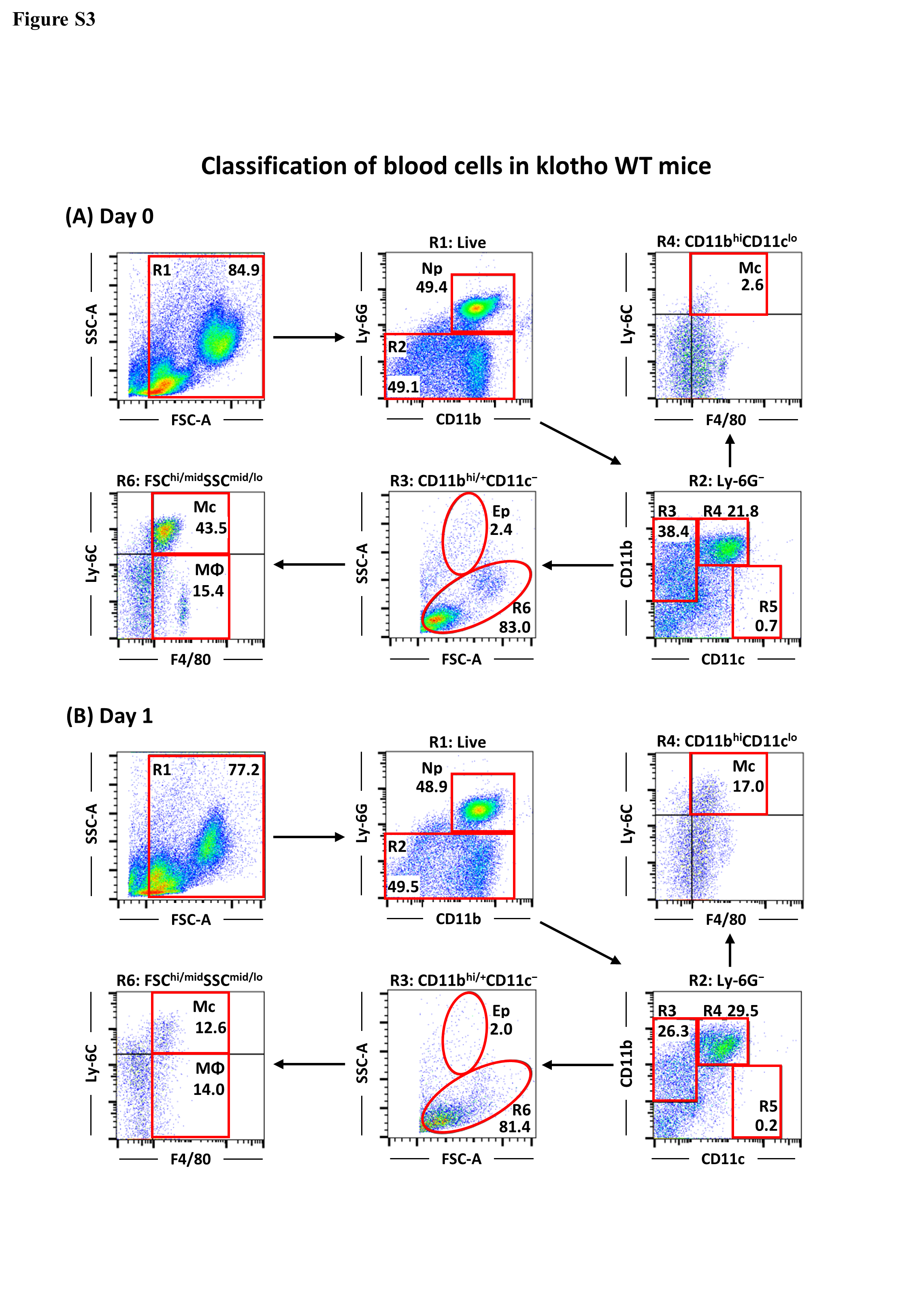

Supplement: Supplementary Figure 3 — Phenotypic analysis of innate immune cells in the blood of Acinetobacter baumannii-infected and uninfected klotho wildtype (WT) mice. Innate immune cells in the blood of A. baumannii-infected and uninfected klotho WT mice were analyzed for their expression of the following cell-surface markers: CD11b, CD11c, Ly-6C, Ly-6G, and F4/80. A representative result of 4-color flow cytometric analysis of the innate immune cell population in blood cells from (A) an uninfected klotho WT mouse and (B) an A. baumannii-infected mouse at 1 day post-infection. Np, Neutrophils (CD11b+Ly-6G+); MΦ, Macrophages (CD11bhigh/+CD11c−Ly-6C+/−Ly-6G−F4/80+ FSChigh/midSSCmid/lo); Mc, Monocytes (CD11bhigh/+CD11clo/−Ly-6ChighLy-6G−F4/80+FSChigh/midSSCmid/lo); Ep, Eosinophils (CD11bhigh/+CD11c−Ly-6G−FSCmidSSChigh); DC, Dendritic cells (CD11blow/−CD11chighLy-6G−). [file Image_3.tif]

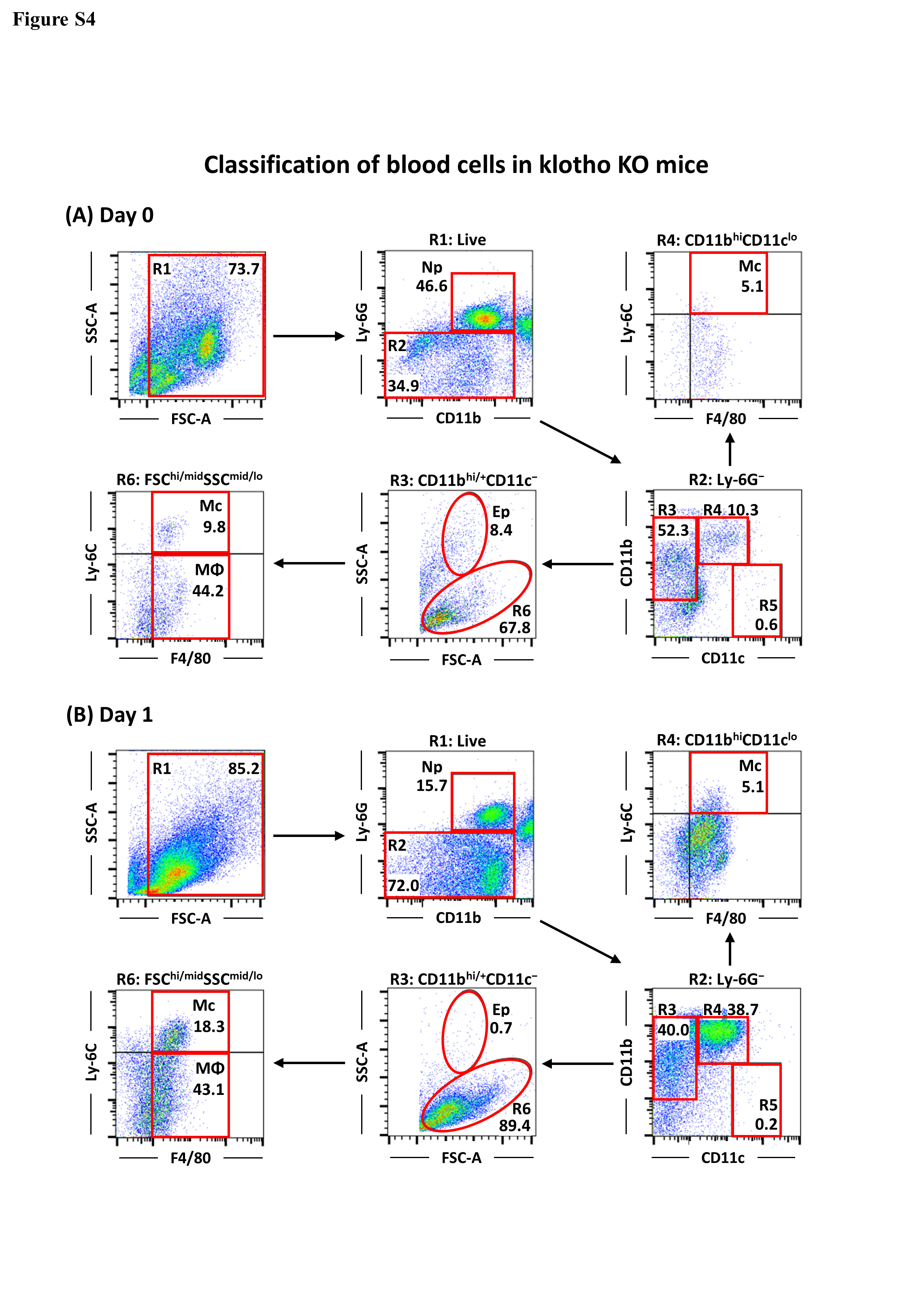

Supplement: Supplementary Figure 4 — Phenotypic analysis of innate immune cells in the blood of Acinetobacter baumannii-infected and uninfected klotho knockout (KO) mice. Innate immune cells in the blood of A. baumannii-infected and uninfected klotho KO mice were analyzed for their expression of the following cell-surface markers: CD11b, CD11c, Ly-6C, Ly-6G, and F4/80. A representative result of 4-color flow cytometric analysis of the innate immune cell population in blood cells from (A) an uninfected klotho WT mouse and (B) an A. baumannii-infected mouse at 1 day post-infection. Np, Neutrophils (CD11b+Ly-6G+); MΦ, Macrophages (CD11bhigh/+CD11c−Ly-6C+/−Ly-6G−F4/80+ FSChigh/midSSCmid/lo); Mc, Monocytes (CD11bhigh/+CD11clo/−Ly-6ChighLy-6G−F4/80+FSChigh/midSSCmid/lo); Ep, Eosinophils (CD11bhigh/+CD11c−Ly-6G−FSCmidSSChigh); DC, Dendritic cells (CD11blow/−CD11chighLy-6G−). [file Image_4.tif]

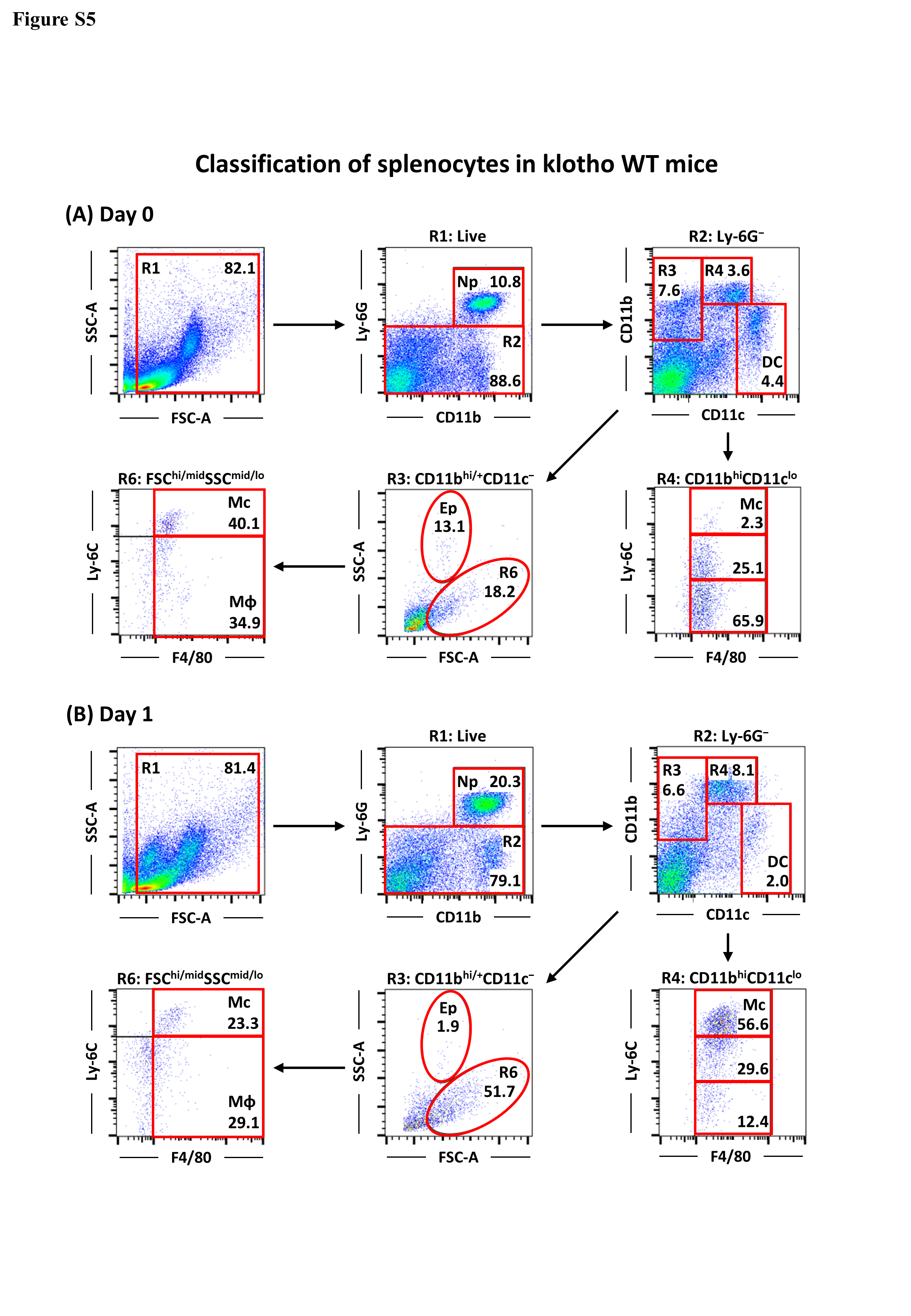

Supplement: Supplementary Figure 5 — Phenotypic analysis of innate immune cells in the spleen of Acinetobacter baumannii-infected and uninfected klotho wildtype (WT) mice. Innate immune cells in the spleen of A. baumannii-infected and uninfected klotho WT mice were analyzed for their expression of the following cell-surface markers: CD11b, CD11c, Ly-6C, Ly-6G, and F4/80. A representative result of 4-color flow cytometric analysis of the innate immune cell population in splenocytes from (A) an uninfected klotho WT mouse and (B) an A. baumannii-infected mouse at 1 day post-infection. Np, Neutrophils (CD11b+Ly-6G+); MΦ, Macrophages (CD11bhigh/+CD11c−Ly-6C+/−Ly-6G−F4/80+ FSChigh/midSSCmid/lo); Mc, Monocytes (CD11bhigh/+CD11clo/−Ly-6ChighLy-6G−F4/80+FSChigh/midSSCmid/lo); Ep, Eosinophils (CD11bhigh/+CD11c−Ly-6G−FSCmidSSChigh); DC, Dendritic cells (CD11blow/−CD11chighLy-6G−). [file Image_5.tif]

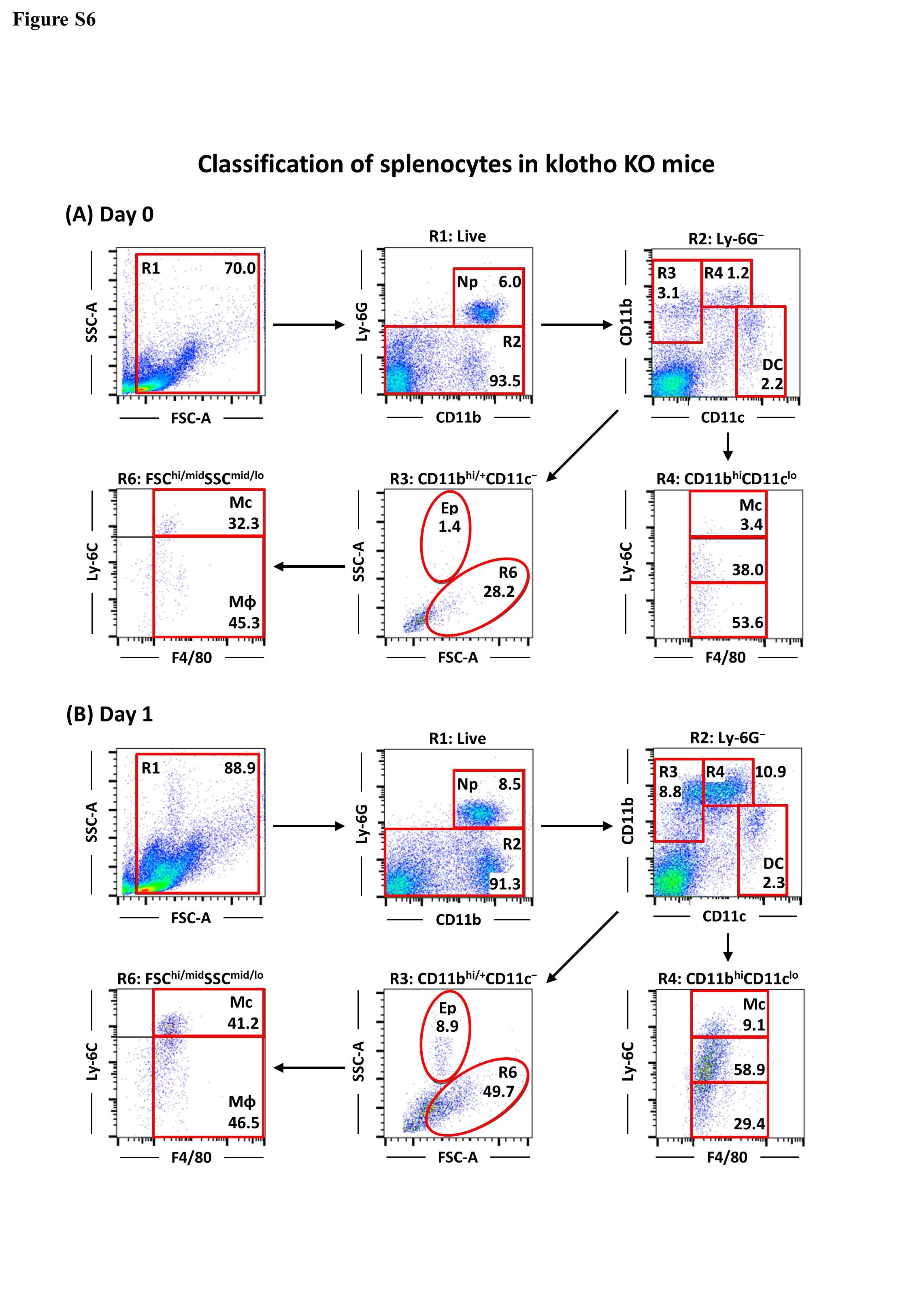

Supplement: Supplementary Figure 6 — Phenotypic analysis of innate immune cells in the spleen of Acinetobacter baumannii-infected and uninfected klotho knockout (KO) mice. Innate immune cells in the spleen of A. baumannii-infected and uninfected klotho KO mice were analyzed for their expression of the following cell-surface markers: CD11b, CD11c, Ly-6C, Ly-6G, and F4/80. A representative result of 4-color flow cytometric analysis of the innate immune cell population in splenocytes from (A) an uninfected klotho WT mouse and (B) an A. baumannii-infected mouse at 1 day post-infection. Np, Neutrophils (CD11b+Ly-6G+); MΦ, Macrophages (CD11bhigh/+CD11c−Ly-6C+/−Ly-6G−F4/80+ FSChigh/midSSCmid/lo); Mc, Monocytes (CD11bhigh/+CD11clo/−Ly-6ChighLy-6G−F4/80+FSChigh/midSSCmid/lo); Ep, Eosinophils (CD11bhigh/+CD11c−Ly-6G−FSCmidSSChigh); DC, Dendritic cells (CD11blow/−CD11chighLy-6G−). [file Image_6.tif]

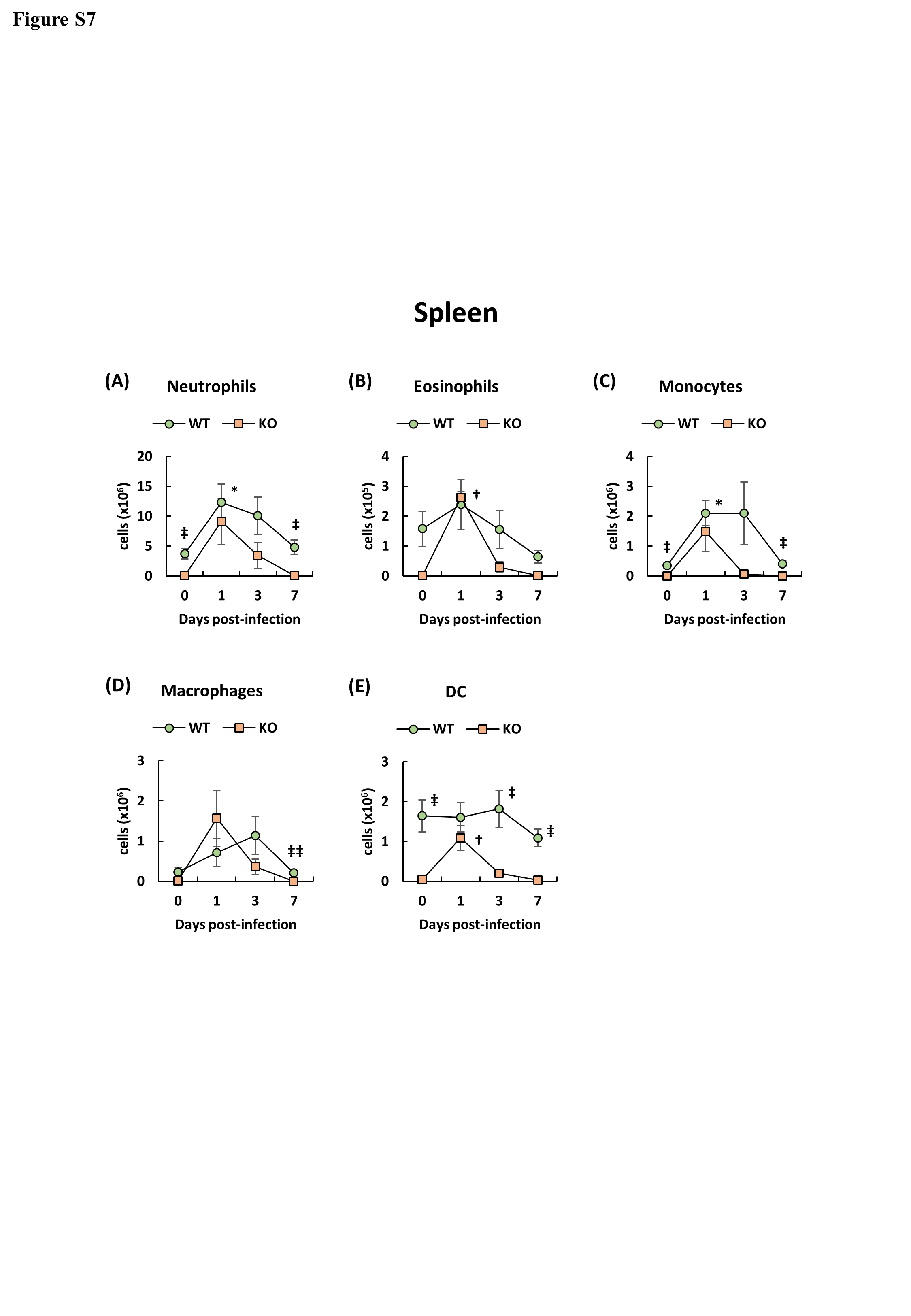

Supplement: Supplementary Figure 7 — Number of innate immune cells in the spleen of klotho wildtype (WT) and knockout (KO) mice before and after infection with Acinetobacter baumannii. Summarized results show the number of (A) neutrophils, (B) eosinophils, (C) monocytes, (D) macrophages, and (E) dendritic cells (DC) in the spleen of A. baumannii-infected klotho WT and KO mice. Line graphs were compiled from 4 independent experiments (n = 16 for WT and n = 16 for KO mice), and represent the mean ± SEM. Asterisks indicate statistically significant differences as follows: ** P < 0.01, * P < 0.05, klotho WT mice before infection vs. klotho WT mice after infection, Student’s t-test; †† P < 0.01, † P < 0.05, klotho KO mice before infection vs. klotho KO mice after infection, Student’s t-test; and ‡‡ P < 0.01, ‡ P < 0.05, klotho WT mice vs. klotho KO mice, Student’s t-test). [file Image_7.tif]
